# Trainable clustering framework for spatial transcriptomics

**DOI:** 10.1093/bioadv/vbag133

**Published:** 2026-05-13

**Authors:** Riasat Azim, Sabab Aosaf, Swakkhar Shatabda, Mohammad Sohel Rahman, Salekul Islam

**Affiliations:** Department of Computer Science and Engineering, United International University, Dhaka, 1212, Bangladesh; Department of Computer Science and Engineering, Bangladesh University of Engineering and Technology, Dhaka, 1000, Bangladesh; Department of Computer Science and Engineering, Brac University, Dhaka, 1212, Bangladesh; Department of Computer Science and Engineering, Bangladesh University of Engineering and Technology, Dhaka, 1000, Bangladesh; Department of Electrical and Computer Engineering, North South University, Dhaka, 1229, Bangladesh

## Abstract

**Summary:**

Spatial transcriptomics (ST) enables high-resolution exploration of tissue architecture by integrating gene expression profiles with spatial information, thereby advancing insights into cellular composition, organization, and interactions. Among ST applications, spatial domain identification is critical for linking gene expression patterns to tissue morphology and analyzing the tissue microenvironment. We introduce a trainable clustering framework that unifies four complementary strategies—ACT, FACT, Scatter, and Ensemble—into a cohesive architecture. By coupling autoencoder-driven feature learning with an Mclust-assisted clustering layer, this framework enables joint optimization of representation and cluster assignments through a trainable loss function. Applied to human DLPFC, mouse brain anterior, and human breast cancer datasets, the proposed framework achieves competitive accuracy in most cases while reliably identifying spatial domains and preserving complex tissue architecture. Additionally, Stereo-seq and Slide-seq datasets are utilized to evaluate cross-platform generalizability and robustness.

**Availability and implementation:**

The proposed framework is implemented in Python. The codes and datasets are available in https://github.com/SababAosaf/FACTSpatialTranscriptomics.

## 1 Introduction

Tissues are composed of diverse cell types whose spatial arrangement is critical for maintaining their biological functions. Recent advancements in spatial transcriptomics (ST) have enabled researchers to study tissue organization and function in unprecedented detail by integrating gene expression with spatial context. ST enables the exploration of cellular composition, structure, and interactions that offer insights into their roles in tissue homeostasis (Meizlish *et al.* 2021) and disease progression.

Despite recent advances, spatial domain clustering, cell type deconvolution, and horizontal cell integration remain major challenges in ST data analysis, as they are critical for elucidating tissue heterogeneity and architecture. Spatial domain identification, referred to as domain-based clustering, is essential for understanding how gene expression patterns correlate with tissue morphology and function.

Current approaches for identifying spatial domains can be broadly categorized into two categories: non-spatial clustering methods and spatial clustering methods. Traditional non-spatial clustering algorithms rely exclusively on gene expression data, treating each sample as independent of its spatial context. In contrast, spatial clustering methods incorporate gene expression data alongside spatial coordinates and morphological features, effectively capturing the inherent spatial dependencies within tissues. This enables spatial clustering methods to capture biologically meaningful patterns more effectively by considering the tissue’s molecular and structural organization.

Spatial domain identification in bioinformatics is formulated as a clustering problem, where spatially-resolved omics profiles are partitioned into distinct, biologically coherent regions based on similarities in their genetic signatures and spatial proximity. To enhance the clustering accuracy and relevance of spatial clusters, supplementary data such as histological images, spatial coordinates of cells or tissues, or expert annotations can be integrated into the gene expression matrix. Spatial data allow for a better understanding of the micro-environmental influences on gene expression. Moreover, expert annotations, such as manual labeling of cell types, can be added to the gene expression matrix. These annotations serve as prior knowledge, guiding the analysis and improving the interpretability of clusters. Integrating histological image data into the gene expression matrix allows for capturing a broader range of cellular and tissue features, potentially uncovering gene expression patterns associated with specific biological traits.

Although originally developed for community detection, the Louvain algorithm (Que *et al.* 2015) has been effectively adapted for domain detection in various applications. It employs a hierarchical clustering approach to identify cell domains of ST. Similarly, the Leiden clustering algorithm (Traag *et al.* 2019) has gained traction for domain annotation, offering improved performance and robustness in identifying distinct clusters within complex datasets. Mclust (Scrucca *et al.* 2023) has also been used for domain annotation and this algorithm focuses on model-based approaches for clustering, classification, and density estimation (Silverman 2018), utilizing finite normal mixture models (McLachlan and Peel 2000). It includes capabilities for estimating parameters through the expectation–maximization (EM) algorithm (Moon 1996) tailored for a range of normal mixture models with diverse covariance structures.

In recent years, deep learning-based spatial domain identification techniques, specifically designed for spatial transcriptomic data, have demonstrated superior performance to traditional statistical approaches. Due to the inherent spatial structure, graph-based representations are commonly used to model spatial relationships between cells or tissue regions. Graph convolutional network-based (Zhang *et al.* 2019) approach SpaGCN (Hu *et al.* 2021) utilized gene expression, spatial location and histology data in ST data analysis and clustering. GraphST (Long *et al.* 2023) another deep learning model uses graph self-supervised contrastive learning (Khosla *et al.* 2020, Liu *et al.*, 2022) for clustering. Meanwhile, DeepST (Xu *et al.* 2022) analyses morphological images and gene expression data to identify spatial patterns. It uses a combination of deep learning and graph neural networks (Wu *et al.* 2021) to create a unified representation of the spatial data.

In particular, BayesSpace (Zhao *et al.* 2021) is a fully Bayesian statistical (Van de Schoot *et al.* 2021) method that uses the information from spatial neighborhoods for resolution enhancement of spatial transcriptomic data and clustering analysis. It effectively addresses challenges related to sparsity and noise commonly encountered in spatial transcriptomic. Furthermore, ConST (Zong *et al.* 2022) uses contrastive learning to generate a low-dimensional embedding for other downstream tasks such as clustering and highly variable gene identification. STAGATE (Dong and Zhang 2022) utilizes graph-attention autoencoder method for ST that learns low-dimensional spot embeddings by jointly modeling gene expression and spatial neighborhood structure. The learned embeddings are then used for downstream clustering to identify spatial domains that respect tissue continuity. GRAPHDeep (Liu *et al.* 2024) utilizes two unsupervised learning modules, VGAE (Kipf and Welling 2016) and DGI (Yin *et al.* 2023) for spatial clustering.

Alongside other deep learning-based approaches, SpaceFlow (Ren *et al.* 2022) used spatially regularized deep graph networks to generate low-dimensional embedding which can be used for downstream tasks. SPAN (ST cell assignment) model assigns cells or spots into known types with prior knowledge of predefined marker genes and spatial information. It then combines a mixture model with an HMRF (hidden Markov random field) to model spatial dependency between neighboring spots and annotate cells or spots. Another approach ScribbleDom (Rahman *et al.* 2023) combines a semi-supervised deep learning-based model with human cognitive ability for spatial domain identification in ST data. Meanwhile, STLearn (Pham *et al.* 2023) uses a two-step clustering procedure. Initially, it generates a broad cluster using the Louvain or KNN clustering method. In the second step, spatial information is used. SC-MEB is an empirical Bayes approach for spatial clustering analysis that leverages a HMRF.

In this study, we present a novel trainable clustering framework comprising four methods—Scatter, ACT, FACT, and an ensemble variant—designed to identify spatial domains or regions that are coherent in both gene expression and histological features. This framework addresses the lack of a universally superior clustering method and leverages its trainable design to adapt to diverse ST datasets. In addition, it integrates an innovative preprocessing technique to enhance data representation and clustering performance. This framework integrates an autoencoder for non-linear dimensionality reduction with a trainable optimization procedure that refines the latent space, enabling effective exploration of the solution space and mitigating the risk of convergence to local minima—a limitation often encountered in traditional optimization techniques. The framework yields robust low-dimensional representations, well suited for accurate spatial domain identification. To validate our approach, we have benchmarked our method on the human dorsolateral prefrontal cortex (DLPFC) (Maynard *et al.* 2021), mouse brain anterior ST (10x Genomics 2020a) and human breast cancer ST (10x Genomics 2020b) dataset. These datasets are selected for their challenging nature, characterized by high dimensionality and complex spatial structure, making them an ideal test case for our method. We have employed multiple benchmarking metrics to comprehensively evaluate the performance of our approach. In most cases, our method outperformed the state-of-the-art techniques.

## 2 Materials and methods

### 2.1 Method overview

**Figure 1:**
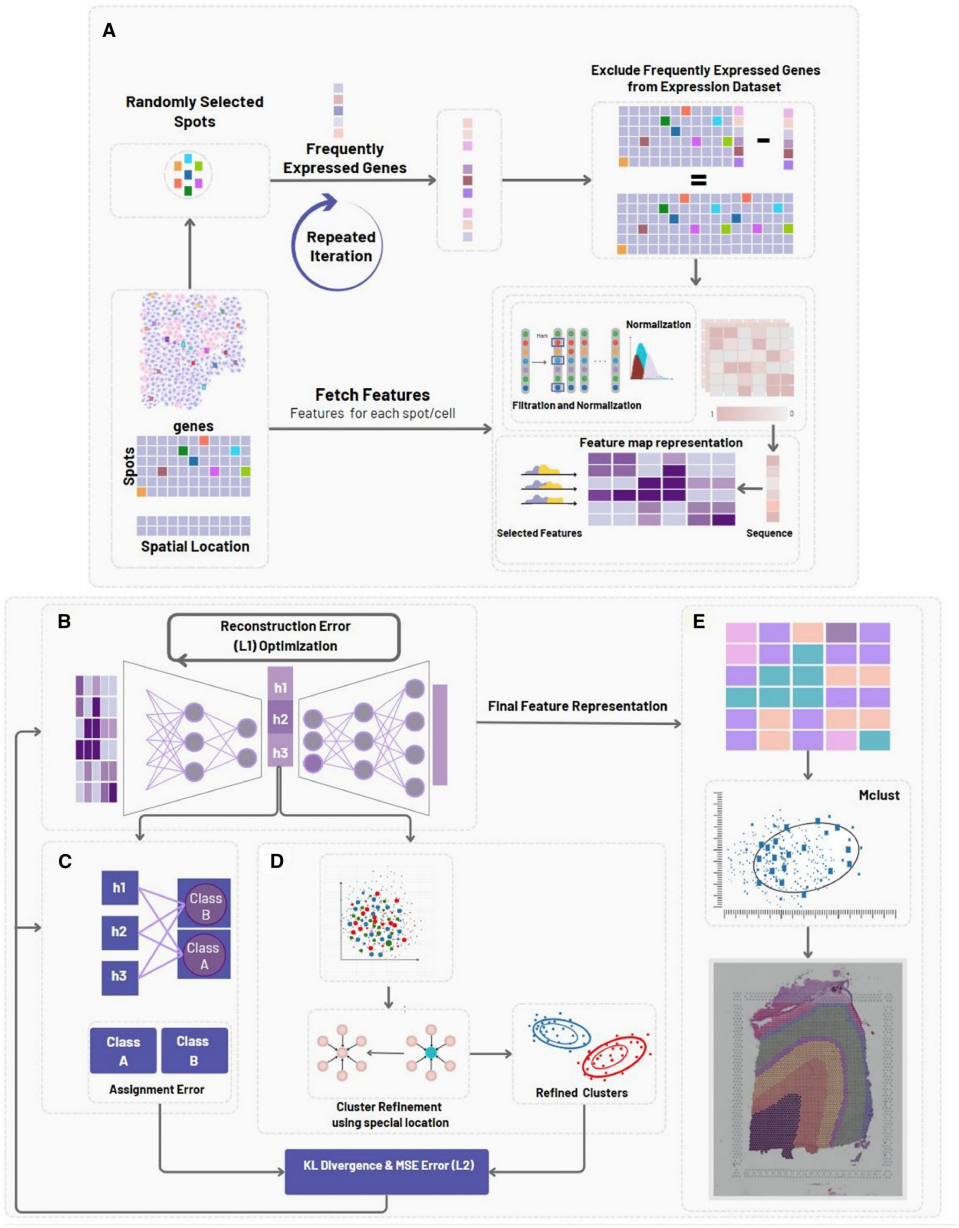
Overall architecture of FACT. (A) Initially, gene-count data with associated spatial coordinates are retrieved. A random subset of spatial spots is iteratively sampled, and genes consistently exhibiting high expression across iterations are identified and removed (we have also proposed ACT method, for which this step is skipped). The resulting filtered and normalized gene-count matrix is used to construct a spatial feature map representation for further analysis. (B) An autoencoder is trained by minimizing the reconstruction error between the input and the reconstructed expression profile. (C) A fully connected clustering layer maps latent embeddings to soft cluster-assignment probabilities in an unsupervised manner. The layer is randomly initialized and not pretrained using ground-truth labels. (D) Using Mclust, class labels are assigned to latent representation (an alternative labeling to C). Which are then refined using spatial location information. The KL divergence between the assigned labels and the clustering layer labeling (from C) is computed. This divergence is then used to further train the autoencoder along with the clustering layer, enhancing the quality of the final feature representations. (E) Finally, the Mclust algorithm is applied on the learned feature representations to identify distinct spatial domains.

The proposed trainable clustering framework for spatial domain identification uses gene expression data and spatial coordinates from tissue. After filtering and normalization, an autoencoder and deep neural network jointly learn latent representations, optimized through clustering and spatial refinement. The final spatial domains are identified by applying Mclust to the learned features. Figure 1 provides a detailed schematic diagram that illustrates the overall architecture of the proposed method. The performance of the framework is evaluated in multiple Visium 10x datasets, including human DLPFC, mouse brain anterior, and human breast cancer, resulting in precise and robust spatial domain identification. Further tests were done for Slide-seqV2 and Stereo-seq datasets. All stages of representation learning, clustering, and spatial refinement are performed in a fully unsupervised manner; ground-truth annotations are used only for *post hoc* evaluation. Also, in our framework, a single shared autoencoder is used to learn latent representations when multimodal inputs were used. In some tests Gene expression features and spatial coordinates are first normalized independently and then concatenated into a unified feature vector, which serves as the input to the autoencoder. No modality-specific encoders are employed; instead, feature fusion is performed through direct concatenation prior to representation learning.

### 2.2 Dataset

The tissue samples comprise gene counts, spatial coordinates, and image-derived features. Gene counts capture expression levels at specific spots or cells, while high-resolution H&E images provide spot-specific information extracted via deep learning models such as ResNet50 (He *et al.* 2016), preserving essential details and reducing dimensionality. For each spatial spot, the normalized gene expression vector is concatenated with its corresponding spatial coordinate features or image embedding to form a joint representation used throughout training (in some cases). To assess spatial domain identification performance, we applied the proposed method to gold-standard ST datasets from multiple platforms. A summary of each dataset used in this study is provided in Table 1.

**Table 1:** Summary of spatial transcriptomics datasets used for benchmarking.

Dataset	Technology	Sample size	Number of slides
DLPFC	10x Visium	3460–4789	12
Mouse brain anterior	10x Visium	2695	1
Human breast cancer	10x Visium	3798	1
Mouse hippocampal formation	Slide-SeqV2	41 786	1
Mouse liver zonation	Stereo-seq	16 211	1

Primarily, our method is applied to The human DLPFC dataset generated by the Visium 10x platform (Maynard *et al.* 2021). It provides high-dimensional gene expression data combined with spatial coordinates, allowing for analysis of cellular organization and gene expression concerning brain anatomy. This dataset is a widely used benchmark for evaluating computational methods in clustering, dimensionality reduction, and spatial domain identification.

For comprehensive benchmarking, we also used the mouse brain anterior ST dataset (10x Genomics 2020a). It was generated with the 10x Genomics Visium platform and contains spatially-resolved transcriptomic profiles of the anterior mouse brain.

For a more comprehensive analysis, the human breast cancer ST dataset (10x Genomics 2020b) is included. The human breast cancer dataset captures transcriptomic heterogeneity within the tumor and stromal tissues. This dataset was generated using the Visium 10x platform.

Additionally, the mouse hippocampal formation dataset from the Slide-seqV2 platform (Stickels *et al.* 2021) and the Mouse Liver Zonation dataset from the Stereo-seq platform (Chen et al. 2022) are used to assess the robustness and cross-platform generalizability of the proposed framework across diverse ST technologies.

### 2.3 Gene filtration

The gene filtration process involves selecting genes with high influence and variability in randomly sampled spots and integrating these influential and highly variable genes into the final set.

The selection of high influence genes involved two different filtration methods that can be used individually or one after another. For the first filtration method, we isolate genes that are most informative for clustering by reducing the influence of broadly expressed “background” genes. We first sample multiple spatial locations within the sample at random and tally gene occurrences across these locations. Genes that consistently show high occurrence across essentially all sampled locations are then iteratively excluded. The reasoning is that genes expressed in many (or most) spatial regions are often linked to housekeeping functions, general viability, or shared cell processes, and therefore tend to provide a weaker signal for separating spatially distinct clusters and are less likely to help in driving meaningful cluster separation. Removing these genes can reduce noise and increase the relative weight of location-enriched signals, which may improve clustering. This is a novel filtration method called scatter filtration as described below.

Let $X\in R^{n\times p}$ be the gene-count (or processed feature) matrix with *n* spots and *p* genes, and let $G_{1}=\{1,\ldots ,p\}$ be the initial gene set. Scatter filtration repeats random spot sampling and removes genes that occur most frequently among the top-expressed genes of sampled spots.

At iteration t=1,…,T, sample a subset of spots


$$S_{t}\subset \{1,\ldots ,n\},\;|S_{t}|=m.$$

For each sampled spot $i\in S_{t}$, define the set of indices of the top-*L* expressed genes


$${\mathrm{Top}}_{L}(x_{i})\subseteq G_{t}.$$

Define the gene-occurrence score (frequency) over the sampled spots:


$$c_{g}^{\left(t\right)}=\sum_{i\in S_{t}}I\left(g\in {\mathrm{Top}}_{L}(x_{i})\right),\;\forall g\in G_{t},$$

where I(·) is the indicator function.

In our implementation, the number of genes removed per iteration is controlled by


$$r=\left\lfloor \frac{\mathrm{knob}}{10}\right\rfloor .$$

where Knob is a user-tunable hyperparameter that controls how intensively genes are removed during each iteration.

Let $R_{t}$ be the set of the *r* most frequently occurring genes:


$$R_{t}={\mathrm{Top}}_{r}\left({\left\{c_{g}^{\left(t\right)}\right\}}_{g\in G_{t}}\right).$$

Update the retained gene set by excluding these high-occurrence genes:


$$G_{t+1}=G_{t}\setminus R_{t}.$$

After *T* iterations, the filtered matrix is


$$X_{\text{filt}}=X_{\left[:,\;G_{T+1}\right]}.$$

For the second filtration method, highly variable genes were identified using the Seurat v3 (Stuart *et al.* 2019) pipeline. Subsequently, only the most influential and highly variable genes were retained, and all other genes were excluded from further analysis, as shown in Fig. 1A.

### 2.4 Normalization

Normalization of feature vectors is a crucial step in ST data analysis, improving the reliability and consistency of downstream analytical methods. To ensure gene expression levels are comparable across cells, raw counts were normalized for each cell based on library size, as shown in Equation (1):


(1) $$X_{\text{norm}}=\frac{X}{\sum X}\cdot s$$

where *X* is the raw count data, ∑X is the total count for each cell, and *s* is the scaling factor.

Thereafter, log normalization is applied to stabilize variance and adjust for smaller values, particularly in datasets exhibiting higher variability. Log normalization is applied as defined by the expression in Equation (2):


(2) $$\text{log}\;1p(x)={\;\text{log}\;}_{e}(x+1)$$

Here, ${\;\text{log}\;}_{e}$ represents the natural logarithm (base *e*), and *x* is the gene expression feature. To standardize the gene expression, the *z*-score normalization is applied, such that each gene expression has a mean of zero and a unit variance, as described in Equation (3):


(3) $$X_{\text{scaled}}=\frac{\;\text{log}\;1p(X)-\mu }{\sigma }$$

where μ is the mean of the data and σ represents the standard deviation of the data.

After scaling, the values in the feature vector are clipped to a maximum threshold to mitigate the influence of outliers that can adversely affect the analysis. Clipping sets all values above a certain threshold to that threshold value. To clip the values within a defined range [a,b] as described in Equation (4):


(4) $$X_{\text{clipped}}=\min(\max(X_{\text{scaled}},a),b)$$

where *a* is the minimum value and *b* is the maximum value.

Following the four-step normalization process as shown in Fig. 1A, the resulting output is a normalized and standardized feature matrix in which gene expression values are appropriately scaled, variance-stabilized, and constrained to mitigate the influence of outliers. This transformation standardizes the gene expression data, improving the accuracy of spatial domain identification in single-cell spatial transcriptomic.

### 2.5 Neighborhood gene expression feature

To incorporate local spatial context into gene expression representations, we define a neighborhood-based gene expression feature for each spatial spot. Let $X\in R^{n\times p}$ denote the normalized gene expression matrix, where *n* is the number of spatial spots and *p* is the number of genes.

For each spot *i*, a spatial neighborhood N(i) is constructed using the *k*-nearest neighbors (kNN) approach based on Euclidean distance between spatial coordinates. Unless otherwise stated, we set k=6, which is consistent with the hexagonal layout of the 10x Visium platform.

To enhance flexibility across ST platforms, the framework is capable of treating the neighborhood parameter as a tunable hyperparameter, allowing adaptation to platform-specific geometry and resolution.

The neighborhood gene expression vector for spot *i* is computed as the mean expression of its neighboring spots:


(5) $$X_{i}^{\left(N\right)}=\frac{1}{\left|N(i)\right|}\sum_{j\in N(i)}X_{j}$$

To obtain a context-aware feature representation, the original gene expression vector of each spot is concatenated with its corresponding neighborhood gene expression vector:


(6) $$Z_{i}=\left[X_{i}\;∥\;X_{i}^{\left(N\right)}\right]$$

where ∥ denotes vector concatenation. This representation captures both intrinsic transcriptional information and local spatial context, and is used as input for downstream clustering and representation learning in the GN-based ablation setting.

### 2.6 Latent representation generation

The transformed feature vectors obtained from the gene filtration and normalization process are subsequently processed by an autoencoder trained to learn a compact latent representation. The latent space is optimized to preserve critical information necessary for accurate reconstruction, while simultaneously ensuring a representation structure that enables effective separation between distinct clusters.

To obtain an optimal latent representation, the training process combines multiple loss functions, including mean squared error (MSE) for reconstruction and KL divergence for assignment error. This latent representation is a lower-dimensional space that preserves the most significant features of the input data. Additionally, a clustering layer is integrated after the autoencoder to enhance cluster segregation.

As shown in Fig. 1B, the autoencoder undergoes a rigorous training process, iteratively refines the model’s ability to reconstruct the input data. The quality of these reconstructions is quantified using reconstruction error, which serves as a loss function in the training process. Specifically, the MSE is utilized to compute the loss.

During training, the autoencoder iteratively updates its parameters to minimize the reconstruction error. At each step, the model adjusts its weights to better approximate the original input of its latent representation. This gradual optimization enhances the model’s capacity to capture the most informative features of input data, leading to increasingly accurate reconstructions over time. After sufficient iterations, the autoencoder is able to efficiently encode the input data into a compact latent representation.

Finally, this iterative training process results in an auto encoder that is capable of learning and encoding the intricate patterns within the data, facilitating various applications such as dimensionality reduction, data denoising, and feature extraction.

### 2.7 Autoencoder and clustering layer architecture

The autoencoder used in the proposed framework follows a symmetric, fully connected architecture designed for non-linear dimensionality reduction. Let $d_{\text{in}}$ denote the input feature dimension after preprocessing. The encoder consists of a fully connected layer, so we have:


$$d_{\text{in}}\to d_{\text{latent}},$$

where $d_{\text{latent}}$ is the dimensionality of the latent representation. In all experiments, we set $d_{\text{latent}}=32$ unless otherwise stated.

The decoder mirrors the encoder architecture:


$$d_{\text{latent}}\to d_{\text{in}}.$$

A fully connected clustering layer is attached to the latent space. This layer maps the latent embedding $z_{i}\in R^{d_{\text{latent}}}$ to a *K*-dimensional vector, where *K* is the predefined number of clusters:


$$d_{\text{latent}}\to K.$$

This layer gives cluster assignments and is trained jointly with the autoencoder using the clustering loss based on KL divergence.

### 2.8 Integration of autoencoder and clustering for latent representation refinement

While the autoencoder weights remain frozen, this prevents updates during subsequent training phases. This step is critical to preserving the learned feature representations and ensuring the autoencoder’s continued ability to generate accurate latent representation. The latent representation is used as the input of the clustering layer. The clustering layer is a fully connected layer that outputs soft cluster-assignment probabilities, trained without ground-truth supervision, with each entity assigned a corresponding cluster membership value. Subsequently, the latent representation is clustered using the Mclust clustering algorithm. The output of the Mclust algorithm is subsequently refined by incorporating information from neighboring spots (cluster memberships are updated based on the current cluster labels of spatially adjacent spots, obtained from unsupervised Mclust assignments). For subsequent stage, two clustering approaches are used: one using a fully connected layer and another based on Mclust as shown in Fig. 1C and D. The KL divergence between these clusters is computed, serving as the loss function for this step. This loss function is used for the back propagation algorithm that trains the clustering layer (enabling unsupervised learning for the clustering layer).

Although the final spatial domains are obtained by applying Mclust to the learned embeddings, the clustering layer is a training-time component that shapes the latent space. Specifically, the clustering layer produces soft assignments that are aligned (via KL divergence) with refined Mclust-based targets during optimization. This additional objective encourages cluster-separable embeddings and reduces representation collapse, thereby improving the suitability of the learned latent space for subsequent model-based clustering with Mclust. The clustering layer is therefore not used as the final labeling mechanism, but as a regularizer that improves representation learning.

### 2.9 Autoencoder fine-tuning with combined reconstruction and clustering loss

In this phase, the weights of the autoencoder are unfrozen, thereby enabling their further optimization during training. This is essential for fine-tuning the entire model. The optimization process simultaneously minimizes two types of errors: the reconstruction error from the autoencoder and the clustering error from the clustering layer. The reconstruction error, calculated using the MSE which measures the difference between the original input data and the reconstructed output from the autoencoder. On the other hand, the clustering error for the clustering layer is computed using the Kullback–Leibler (KL) divergence (Shlens 2014) as presented in Equation (7):


(7) $$D_{\text{KL}}(P∥Q)=\sum_{i}P(i)\;\text{log}\;\frac{P(i)}{Q(i)}$$

where *Q* is resultant probability distribution and *P* is reference probability distribution for *i* event.

KL divergence measures the divergence between the distribution of latent representations and a target distribution, thereby facilitating more effective clustering of the latent embeddings. Both errors are weighted according to their importance in the overall model training process and then combined into a single loss function. This composite loss function guides the training of the autoencoder and clustering layer, ensuring the model becomes efficient at both reconstructing input data and accurately clustering the latent representations.

### 2.10 Joint optimization of reconstruction and clustering objectives

The representation learning component of the proposed framework is implemented as a **linear autoencoder** augmented with a clustering head. Let $X\in R^{n\times d}$ denote the input feature matrix, where *n* is the number of spatial spots and *d* is the input feature dimension.

The encoder maps the input features into a latent space:


(8) $$Z=XW_{1},$$

where $W_{1}\in R^{d\times d_{z}}$ and $Z\in R^{n\times d_{z}}$ are the latent embedding. The decoder reconstructs the input from the latent representation:


(9) $$\hat{X}=ZW_{2},$$

where $W_{2}\in R^{d_{z}\times d}$. The autoencoder is trained by minimizing the mean squared reconstruction error:


(10) $$L_{\text{rec}}=\frac{1}{n}\sum_{i=1}^{n}||X_{i}-{\hat{X}}_{i}|{|}_{2}^{2}.$$

#### 2.10.1 Clustering layer and soft assignments

A fully connected clustering layer is attached to the latent embedding *Z* to produce cluster-assignment scores:


(11) $$S=ZW_{3},$$

where $W_{3}\in R^{d_{z}\times K}$ and *K* is the predefined number of clusters. The scores are converted into soft cluster-assignment probabilities using a softmax function:


(12) $$q_{ik}=\frac{\;\text{exp}(S_{ik})}{\sum_{k^{\prime }=1}^{K}\;\text{exp}\;(S_{ik^{\prime }})}.$$

In parallel, Mclust is applied to the latent embeddings *Z* to obtain cluster labels based on a Gaussian mixture model. These labels are further refined using spatial neighborhood. The refined labels are then converted into a one-hot encoded matrix $Y\in {\left\{0,1\right\}}^{n\times K}$, which serves as a reference distribution for clustering supervision.

The discrepancy between the soft assignments $Q=\{q_{ik}\}$ produced by the clustering layer and the refined Mclust targets *Y* is measured using the KL divergence:


(13) $$L_{\text{clust}}=\text{KL}(Y∥Q).$$

#### 2.10.2 Joint optimization

The linear autoencoder and clustering layer are trained jointly by minimizing a weighted combination of the reconstruction and clustering losses:


(14) $$L_{\text{total}}=\lambda_{\text{rec}}L_{\text{rec}}+\lambda_{\text{clust}}L_{\text{clust}},$$

where $\lambda_{\text{rec}}$ and $\lambda_{\text{clust}}$ control the tradeoff between reconstruction fidelity and clustering consistency. During training, the optimization proceeds in stages, beginning with reconstruction-focused learning, followed by clustering alignment and final joint refinement, as described in the implementation.

#### 2.10.3 Pipeline design justification

We adopted a two-stage optimization strategy because applying a clustering objective from the beginning can be unstable when the latent space has not yet learned a meaningful representation of the input. The first stage therefore focuses only on reconstruction, allowing the autoencoder to learn a stable and information-preserving embedding. Once this embedding has been established, the clustering objective is introduced to refine the latent space rather than define it from scratch. This staged design separates representation formation from cluster refinement, which improves optimization stability and reduces the risk that early clustering signals distort the underlying biological structure.

We also retain the reconstruction objective during the second stage because clustering alone does not ensure that the learned embedding remains faithful to the original data. If the model was optimized only for clustering, it could move toward embeddings that are easier to separate but less representative of the transcriptomic and spatial information contained in the input. Keeping the reconstruction term therefore acts as a safeguard against this drift and ensures that the latent representation remains both informative and cluster-friendly. In this sense, the combined objective is intended to balance two complementary goals: preserving data fidelity and improving cluster separability.

The clustering layer is introduced as a training-time regularizer rather than as the final labeling mechanism. Its purpose is to provide an additional signal that shapes the latent space into a more organized and cluster-separable structure during learning. Final labels are still obtained with Mclust so that the downstream clustering procedure remains fixed across variants, making it possible to attribute any performance gain to improved representation learning rather than to a change in the final clustering algorithm. Using refined Mclust assignments as reference targets is also appropriate in the ST setting, because the refinement step incorporates neighborhood information and therefore better reflects the expectation that true spatial domains should be locally coherent rather than spatially fragmented.

Within this design, KL divergence is a suitable choice for training the clustering layer because the layer produces soft cluster-assignment probabilities. In this setting, the goal is not simply to match numerical values, but to align one probability distribution with another. KL divergence is well suited for that purpose because it directly measures how far the predicted assignment distribution is from the refined target distribution. This makes it more appropriate than a standard regression-style loss for guiding soft cluster memberships. In addition, KL divergence allows the model to remain uncertain during the early stages of joint training, when cluster boundaries may still be ambiguous, while progressively encouraging sharper and more consistent assignments as the latent space improves. As a result, the clustering term provides a principled distribution-level supervision signal that complements the reconstruction objective and helps produce embeddings that are more suitable for downstream clustering.

Overall, the design is intended to first learn a stable latent representation, then refine that representation using a clustering-aware objective without sacrificing reconstruction quality, and finally obtain the spatial domains using a fixed model-based clustering step. This makes the clustering layer a mechanism for improving the geometry of the latent space during training, rather than a replacement for the final clustering procedure.

### 2.11 Metaheuristic training approach

The metaheuristic training approach can be employed to update the weights of the autoencoder, not during every iteration but after a predefined number of iterations. The latent representation generated by the autoencoder is then clustered using a clustering algorithm, such as DBSCAN. The resulting clustering is evaluated using an ASW score that does not require ground truth. This score serves as the fitness function that guides the metaheuristic process. The hill-climbing approach is used to steer the process toward better fitness. However, a higher fitness or ASW score does not necessarily indicate that the new clustering is closer to the ground truth, which is the main shortcomings of this approach.

The metaheuristic training is used only as an ablation (hill-climbing/ASW) to explore whether optimizing ASW improves results; it is not included in the final model.

### 2.12 Cluster refinement

The latent representation is clustered using Mclust. The results are then refined through a post-processing step aimed at enhancing the consistency of the clustering labels. For each individual spot, an evaluation is conducted to determine the clustering labels of its neighboring spots based on similarity. If the majority of these neighboring spots had a different clustering label compared to the spot in interest, the label of that spot is adjusted to match the majority label of its neighbors. This refinement process ensures that the clustering labels are more cohesive and better reflect the overall structure and patterns within the data, resulting in a more accurate and reliable clustering outcome. After this step, the cluster assignment for each spot is determined as shown in Fig. 1E. Importantly, this refinement step operates solely on predicted cluster labels and spatial proximity, without using any manual annotations or ground-truth information.

### 2.13 Final feature construction

The proposed framework supports multiple feature combinations, which are systematically evaluated through an ablation study. However, the final configuration adopted for the proposed model (ACT, FACT, and the Ensemble variants) uses **gene expression profiles and spatial coordinates only**. Spatial features are integrated through spatially informed refinement during clustering rather than direct concatenation. A single shared autoencoder is trained on the gene expression matrix, while spatial coordinates are incorporated during clustering refinement and neighborhood-based post-processing. Image-derived morphological features extracted from H&E-stained tissue sections are evaluated only in the ablation study and are not included in the final model.

Notably, adding an extra modality does not necessarily improve clustering, especially when it is noisy or weakly aligned with the biological domains of interest. Moreover, it may introduce spurious signals that degrade performance.

This design choice is motivated by empirical results showing that image-only or image-augmented feature combinations consistently underperform gene expression–centric configurations and may introduce noise when directly merged with transcriptomic features.

#### 2.13.1 Ensemble construction

Let M denote the candidate set of methods (e.g. M={STAGATE,GraphST,Scatter,ACT,FACT}). For each sample (slide) *s* and each method m∈M, we compute five unsupervised quality metrics: average silhouette width (ASW) (Batool and Hennig 2021), partition accuracy score (PAS) (Hubert and Arabie 1985, Jain *et al.* 1999), clustering heterogeneity and order statistic (CHAOS) (Hu *et al.* 2024), Moran’s *I* (MoranI) (Moran 1950), and Geary’s *C* (GearyC) (Geary 1954). These metrics capture complementary aspects of clustering quality: ASW reflects separation/compactness, PAS and CHAOS reflect structural stability/disorder, and MoranI/GearyC quantify spatial autocorrelation. As different methods may perform optimally on different slides, we select a method per slide based solely on unsupervised criteria and evaluate the resulting clustering using ARI, which is not used for selection.

#### 2.13.2 Per-slide normalization

Metrics have different scales, so for each slide *s* we normalize each metric across methods. For a metric *k* and method *m*, let $x_{m,k}(s)$ be the raw value. Define


(15) $$z_{m,k}(s)=\frac{x_{m,k}(s)-\underset{m^{\prime }\in M}{\min}x_{m^{\prime },k}(s)}{\underset{m^{\prime }\in M}{\max}x_{m^{\prime },k}(s)-\underset{m^{\prime }\in M}{\min}x_{m^{\prime },k}(s)+\varepsilon },$$

where ε is a small constant to avoid division by zero. For metrics to be minimized (PAS, CHAOS, GearyC), we use the benefit form


(16) $$z_{m,k}(s)\leftarrow 1-z_{m,k}(s),\;k\in \{\text{PAS},\text{CHAOS},\text{GearyC}\},$$

so that in all cases larger $z_{m,k}(s)$ indicates better quality.

#### 2.13.3 Ensemble selection (balanced reference-point rule)

A selector that optimizes a single metric (e.g. always maximizing ASW) may be brittle when that metric performs sub-optimally on a given slide or when extreme spatial smoothing or fragmentation artificially inflates certain metrics.

To obtain a robust compromise, we define a per-slide reference profile that prefers strong separation and spatial coherence, while avoiding degenerate extremes in stability/disorder metrics. Specifically, define the reference components


(17) $$\begin{array}{c} t_{\text{ASW}}(s)=\underset{m\in M}{\max}z_{m,\text{ASW}}(s),\\ t_{\text{MoranI}}(s)=\underset{m\in M}{\max}z_{m,\text{MoranI}}(s),\\ t_{k}(s)={\text{median}}_{m\in M}z_{m,k}(s),\;k\in \{\text{PAS},\text{CHAOS}, \end{array}$$

(18) $$\begin{array}{c} \text{GearyC}\}. \end{array}$$

We then select the method whose normalized metric vector is closest to this reference profile:


(19) $$m^{*}(s)=\arg\underset{m\in M}{\min}\sum_{k\in K}{\left(z_{m,k}(s)-t_{k}(s)\right)}^{2},$$

where K={ASW,PAS,CHAOS,MoranI,GearyC}. The ensemble output for slide *s* is the clustering produced by $m^{*}(s)$. When ground-truth labels are available, we report the corresponding ARI for evaluation; ARI is *never* used in the selection step.

We also employ metric-specific ensemble selection strategies. In the ASW-based, Ensemble (ASW), the clustering result with the highest ASW value is selected. In the PAS-based, Ensemble (PAS), the result achieving the minimum PAS value is chosen. Similarly, the CHAOS-based, Ensemble (CHAOS) selects the result with the minimum CHAOS score. Moreover, the combined ensemble method discussed in this section is defined as Ensemble.

The Ensemble does not use fixed manual weights for the five metrics. Instead, for each slide we first normalize all metrics across candidate methods, convert PAS, CHAOS, and Geary’s *C* to a common benefit scale, and then select the method closest to a balanced reference profile. This reference favors strong separation and spatial coherence through ASW and Moran’s *I*, while avoiding degenerate over-smoothed or over-fragmented solutions by using balanced targets for PAS, CHAOS, and Geary’s *C*. Metric misalignment remains a general risk in model selection, which is why we use a balanced multi-metric rule rather than relying on any single criterion alone.

### 2.14 Performance metrics

To comprehensively assess the performance of spatial domain clustering, we evaluate clustering accuracy using multiple performance metrics. For a robust comparison with state-of-the-art methods, we have employed normalized mutual information (NMI), adjusted rand index (AR), Geary’s *C* score (Geary 1954), Moran’s *I* score (Moran, 1950), ASW (Batool and Hennig 2021), PAS score, CHAOS score, completeness score, and Homogeneity score.

#### 2.14.1 The ARI

The ARI is used for analysing the similarity between two clustering (Santos and Embrechts 2009). It evaluates pairs of samples, checking if they are assigned to the same or different clusters in both the predicted and true clusterings. The ARI value is 0.0 for random labeling and exactly 1.0 when the clusterings are identical


(20) $$\text{ARI}=\frac{\sum_{ij}\left(\begin{array}{c} n_{ij}\\ 2 \end{array}\right)-\left[\sum_{i}\left(\begin{array}{c} a_{i}\\ 2 \end{array}\right)\sum_{j}\left(\begin{array}{c} b_{j}\\ 2 \end{array}\right)\right]/\left(\begin{array}{c} n\\ 2 \end{array}\right)}{\frac{1}{2}\left[\sum_{i}\left(\begin{array}{c} a_{i}\\ 2 \end{array}\right)+\sum_{j}\left(\begin{array}{c} b_{j}\\ 2 \end{array}\right)\right]-\left[\sum_{i}\left(\begin{array}{c} a_{i}\\ 2 \end{array}\right)\sum_{j}\left(\begin{array}{c} b_{j}\\ 2 \end{array}\right)\right]/\left(\begin{array}{c} n\\ 2 \end{array}\right)}$$

- *i*: index of a cluster in the ground-truth partition.
- *j*: index of a cluster in the predicted partition.
-
$n_{ij}$ : number of samples simultaneously assigned to cluster *i* in the ground-truth partition and cluster *j* in the predicted partition.
-
$a_{i}=\sum_{j}n_{ij}$ : size of the *i*th ground-truth cluster.
-
$b_{j}=\sum_{i}n_{ij}$ : size of the *j*th predicted cluster.
-
$\left(\begin{array}{c} x\\ 2 \end{array}\right)$ : denotes the binomial coefficient, which is the number of ways to choose two items from *x* items.

#### 2.14.2 Normalized mutual information

Normalized Mutual Information (NMI) is also used for assessing the similarity between two clustering results (Kvålseth 2017). It compares the shared information between the clustering labels and the true labels, normalized by the average information in each set. NMI score of 1 indicates perfect agreement and 0 indicates no mutual information


(21) $$\text{NMI}(U,V)=\frac{2\times I(U;V)}{H(U)+H(V)}$$

(22) $$I(U;V)=\sum_{u\in U}\sum_{v\in V}P(u,v)\;\text{log}\;\left(\frac{P(u,v)}{P(u)P(v)}\right)$$

(23) $$H(U)=-\sum_{u\in U}P(u)\;\text{log}\;P(u)$$

(24) $$H(V)=-\sum_{v\in V}P(v)\;\text{log}\;P(v)$$

- *U*: the first clustering.
- *V*: the second clustering.
-
I(U;V) : mutual information between clusterings *U* and *V*.
-
H(U) : entropy of clustering *U*.
-
H(V) : entropy of clustering *V*.
-
P(u,v) : joint probability of a pair of clusters *u* and *v*.
-
P(u) : probability of cluster *u* in clustering *U*.
-
P(v) : probability of cluster *v* in clustering *V*.
-
log  : natural logarithm.

Detailed descriptions of Geary’s *C* score, Moran’s *I* score, ASW, PAS score, CHAOS score, completeness score, and homogeneity score can be found in Supplementary Equations section of the Supplementary Document 1, available as supplementary data at *Bioinformatics Advances* online.

## 3 Result analysis

In this section, we present the results of our comprehensive analysis across multiple ST datasets, including human breast cancer, DLPFC, and mouse brain anterior. To evaluate the framework’s performance, assessing biological signal detection and domain structure identification is crucial. A range of quantitative metrics is employed for this purpose, including ARI, NMI, Geary’s *C*, Moran’s *I*, ASW, PAS score, CHAOS score, completeness, and homogeneity. Benchmarking against state-of-the-art methods highlights the strengths and limitations of each approach. Additionally, computational efficiency is assessed to determine the method’s suitability for practical use. An ablation study is further performed to evaluate the contribution of different features and components to overall performance.

### 3.1 Ablation study

We conduct a controlled ablation study on the human DLPFC benchmark by running each variant on all 12 tissue sections and evaluating agreement with manual cortical-layer annotations using ARI (Table 3). All variants are evaluated in a fully unsupervised manner: ground-truth labels are used *only* for *post hoc* ARI computation and are never used during feature learning, refinement, clustering, or method selection. To isolate the effect of the ablated component (input modality and/or training strategy), we keep the downstream label assignment procedure fixed across variants: *final labels are always obtained by applying Mclust to the learned latent embeddings*. Unless otherwise stated, all autoencoder-based variants share the same symmetric fully connected autoencoder backbone and latent dimension ($d_{\text{latent}}=32$), and the same gene preprocessing pipeline (normalization and variance-stabilization) so that ARI differences reflect the feature set and optimization strategy rather than implementation artifacts. For variants that require neighborhood context, we construct a spatial kNN graph from spot coordinates and use number of neighbors, k=6.

This selection is consistent with the spatial organization of spots in the 10x Visium platform, which follows a hexagonal lattice structure. In a hexagonal arrangement, each spot typically has six immediate adjacent spots. Hence, setting k=6 enables the neighborhood graph to reflect the intrinsic adjacency relationships present in the Visium architecture. This ensures that the spatial connectivity used in the analysis is aligned with the underlying physical layout of the tissue spots, thereby providing a more realistic representation of local spatial interactions. For other spatial configurations, *k* can be treated as a tunable parameter: square-like layouts may favor 4- or 8-neighbor graphs, while cell-resolved platforms often benefit from radius-based or Delaunay-type neighborhood graphs.

#### 3.1.1 Description of variants evaluated

The details of the variants included in our experiments are given below. For details, please see Table 2.

- G: Gene expression only → autoencoder → Mclust.
- GS: Gene expression + spatial coordinates (concatenation after independent normalization) → autoencoder → Mclust.
- GN: Gene expression + neighborhood mean expression (k=6) concatenated with own expression → autoencoder → Mclust.
- I: Image-only (fixed pretrained CNN embeddings) → autoencoder → Mclust.
- IG, IS, IGS: Early fusion baselines using concatenation of (I) with gene/spatial features → autoencoder → Mclust.
- GD, GSD: Metaheuristic-trained autoencoder variants (hill climbing guided by an unsupervised compactness metric, ASW from DBSCAN) → Mclust.
- Scatter: Scatter filtration (remove highly occurring genes from randomly sampled spots) + autoencoder → Mclust.
- ACT: Autoencoder + clustering layer trained with a KL divergence objective against *refined Mclust-based targets*; final labels still via Mclust.
- FACT: Scatter filtration integrated into ACT (filtration + joint representation − clustering optimization + refinement) → final labels via Mclust.
- Ensemble: Execute and evaluate {Scatter, ACT, FACT, GraphST, STAGATE}, subsequently select one output per slide using combination of unsupervised quality metrics (ASW, PAS, CHAOS, Moran’s *I*, and Geary’s *C*).

**Table 2:** Overview of methods and feature combinations used for spatial transcriptomics clustering.

Method name	Features	Metaheuristic	Technique
G	Gene expression	No	An autoencoder generates latent representations from the selected features, generating clusters using the Mclust algorithm
I	Image	No	
IG	Gene expression	No	
	Image.		
IS	Image	No	
	Spatial location		
IGS	Image	No	
	Gene expression		
	Spatial location		
GS	Gene expression	No	
	Spatial location		
GN	Gene expression	No	
	Neighbors gene expression		
	(mean of *k*NN neighbors, k=6)		
GD	Gene expression	Yes	The autoencoder is trained using a hill-climbing metaheuristic, optimizing the clustering quality of its latent representations, measured by the average silhouette width (ASW) from DBSCAN, followed by final cluster assignment using Mclust
GSD	Gene expression	Yes	
	Spatial location		
Scatter	Gene expression	No	Highly occurring genes from randomly selected spots are removed, and the gene-count features are used to train an autoencoder
ACT	Gene expression	No	The autoencoder is initially trained on the gene-count matrix, followed by joint optimization with a clustering layer that incorporates a novel refinement strategy leveraging spatial coordinates and final cluster assignment via Mclust
	Spatial location		
FACT	Gene expression	No	The filtration strategy from Scatter is integrated into the ACT framework to enhance feature selection
	Spatial location		
Ensemble	Gene expression	No	FACT, ACT, Scatter, GraphST, and STAGATE are executed independently, and the result is selected using a balanced multi-metric selector
	Spatial location		

**Table 3:** Clustering performance (ARI) of different method variants tested on 12 DLPFC slides.^a^

	151507	151508	151509	151510	151669	151670	151671	151672	151673	151674	151675	151676	**Mean **±** SD**
G	0.53	0.00	0.43	0.47	0.40	0.39	0.60	0.62	0.66	**0.59**	0.47	0.64	0.483 ± 0.179
GS	0.55	0.41	0.47	0.60	0.41	0.39	0.61	0.59	0.62	0.46	0.58	0.63	0.527 ± 0.092
I	0.10	0.17	0.15	0.15	0.17	0.14	0.18	0.07	0.06	0.12	0.12	0.13	0.130 ± 0.038
IG	0.13	0.17	0.16	0.15	0.11	0.20	0.14	0.10	0.18	0.23	0.12	0.22	0.159 ± 0.042
IS	0.14	0.16	0.15	0.14	0.16	0.15	0.18	0.07	0.06	0.12	0.12	0.13	0.132 ± 0.036
IGS	0.16	0.17	0.16	0.15	0.18	0.17	0.16	0.16	0.24	0.21	0.16	0.15	0.172 ± 0.027
GSD	0.48	0.33	0.45	0.50	**0.54**	0.38	0.59	0.59	0.60	0.44	0.42	0.42	0.478 ± 0.088
GD	0.52	0.49	0.47	0.42	0.32	0.39	0.55	0.61	0.64	0.43	0.46	**0.64**	0.495 ± 0.101
GN	0.49	0.33	0.50	0.52	0.33	0.37	0.60	0.62	0.65	0.58	0.55	0.52	0.505 ± 0.109
Scatter	0.47	0.40	0.48	0.38	0.40	0.38	0.47	**0.73**	0.66	0.55	0.46	0.43	0.484 ± 0.111
ACT	0.49	0.34	0.48	0.35	0.37	0.39	**0.62**	0.58	0.66	0.58	0.32	0.37	0.463 ± 0.122
FACT	0.55	0.31	**0.51**	0.44	0.39	0.41	0.64	0.61	0.67	0.47	0.25	0.26	0.459 ± 0.143
Ensemble	**0.59**	**0.49**	0.48	**0.51**	0.40	**0.51**	0.47	0.61	**0.67**	0.43	**0.57**	0.43	**0.513** ± **0.082**

a The final column reports the mean ± SD across the 12 slides.

#### 3.1.2 Ablation results

Table 3 shows that gene-expression–centric representations provide the dominant signal for cortical-layer recovery, while image-only baselines underperform. Specifically, the image-only methods yield low ARI across slides (mean ARI: I =0.130, IS =0.132), indicating that morphology embeddings alone are insufficient to recover DLPFC laminar structure. Early fusion with image features provides only modest gains (IG mean ARI =0.159, IGS mean ARI =0.173) and remains substantially below gene-centric configurations, suggesting that naive concatenation can introduce nuisance variation that competes with transcriptomic laminar signals.

In contrast, incorporating spatial context alongside gene expression produces the strongest and most stable ablation performance. Adding spatial coordinates (GS) improves the mean ARI from 0.483 (G) to 0.527 (GS) and substantially reduces variability across sections (std. 0.179 for G versus 0.092 for GS), reflecting improved robustness under slice-to-slice heterogeneity. This effect is especially pronounced on difficult sections: for slide 151508, performance increases from ARI =0.00 (G) to ARI =0.41 (GS), and on other representative slides GS improves over G on 151510 (0.60 versus 0.47) and 151675 (0.58 versus 0.47). Neighborhood augmentation (GN) is also competitive (mean ARI =0.505), improving over G on multiple slides such as 151509 (0.50 versus 0.43), 151510 (0.52 versus 0.47), and 151675 (0.55 versus 0.47), consistent with the biological expectation that cortical layers are transcriptionally distinct *and* spatially contiguous. Metaheuristic variants (GD/GSD) can yield strong performance on particular sections (e.g. GD reaches 0.49 on 151508 compared to 0.00 for G), but their benefits are not uniformly expressed across all slides, highlighting the importance of a stable spatially informed feature configuration.

Among the proposed framework variants, Scatter, ACT, and FACT exhibit complementary strengths across heterogeneous slides. Scatter achieves the highest single-slide ARI in Table 3 on slide 151672 (ARI =0.73), indicating that filtration of broadly expressed/background genes can substantially sharpen separability in certain sections. FACT reaches strong performance on slides with clear laminar structure (e.g. 151671: 0.64; 151673: 0.67), and on slide 151673 both FACT and the Ensemble attain the best ARI (0.67). Overall, the Ensemble provides the best average ARI among the proposed variants (mean ARI =0.513), consistent with increased robustness via integrating complementary strategies. Because the Ensemble selects outputs using unsupervised compactness metrics, it can underperform the best ARI-achieving single method on some slides (e.g. 151671: Ensemble 0.47 versus ACT 0.62 and FACT 0.64; 151672: Ensemble 0.61 versus Scatter 0.73), which is expected when unsupervised selection criteria and annotation-based metrics are not perfectly aligned.

Based on the ablation results, gene expression combined with spatial information yields strong and stable performance across datasets; therefore, this feature set was selected as the final configuration for the proposed framework. This conclusion is supported quantitatively by the consistent gains and reduced variance observed when spatial context is incorporated with gene expression (GS: mean ARI 0.527, std. 0.092) relative to gene-only learning (G: mean ARI 0.483, std. 0.179), as well as by the large improvement on challenging slices such as 151508 (0.41 versus 0.00).

The ablation study shows that ACT, which adds a clustering layer with a KL divergence objective, improves ARI on challenging sections (151508: 0.00 → 0.34; 151509: 0.43 → 0.48; 151671: 0.60 → 0.62) while matching performance on high-signal slides. Method G lacks this layer. These results indicate the clustering layer refines the latent space to aid model-based clustering rather than directly producing final labels.

The hill-climbing metaheuristic is used only in the GD/GSD ablation variants and is not part of the final ACT, FACT, or Ensemble framework. This distinction is important because the metaheuristic provides only a limited or inconsistent ARI benefit while substantially increasing runtime. Specifically, on the DLPFC benchmark, GD improves mean ARI only slightly relative to G (0.495 versus 0.483) while increasing mean runtime from 52.25 to 252.94 s per slide. Likewise, GSD underperforms GS in mean ARI (0.478 versus 0.527) while increasing mean runtime from 48.95 to 270.75 s per slide (Tables 6–9, available as supplementary data at *Bioinformatics Advances* online). It is noteworthy that metaheuristic optimization was not retained in the final framework because its cost–benefit tradeoff was unfavorable.

### 3.2 Application on DLPFC dataset

The DLPFC ST dataset captures gene expression patterns in the human DLPFC, generated using the 10x Genomics Visium platform, it provides spatially-resolved transcriptomic data across anatomically defined cortical layers. This dataset is widely used for investigating brain architecture and serves as a benchmark for spatial domain detection methods. DLPFC dataset comprises spatially-resolved transcriptomic profiles from 12 DLPFC tissue sections. It is important to note that performance on the DLPFC dataset is inherently slice-dependent. Certain tissue sections exhibit clearer laminar separation, for which the Ensemble strategy provides substantial benefit, while others contain irregular layer boundaries and higher gene expression overlap, favoring specific single variants. Therefore, the Ensemble method is designed to optimize robustness across the full dataset rather than guarantee superior performance on every individual slice.

The proposed framework, a trainable loss optimization-based spatial domain identification technique, was applied to the DLPFC dataset. The model was evaluated across all 12 DLPFC slides to assess its domain identification performance using various clustering metrics.

**Figure 2:**
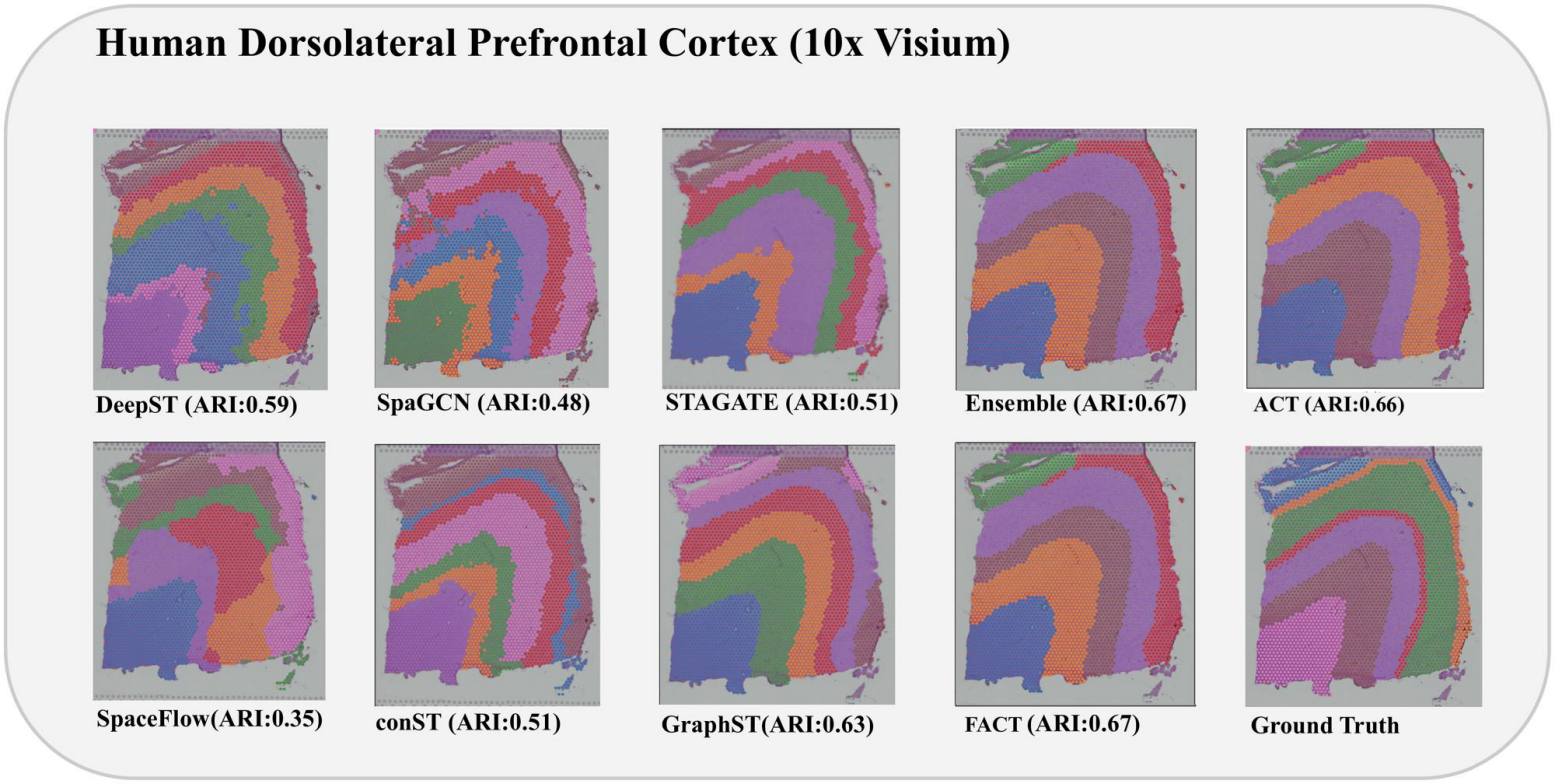
Comparison of spatial domain identification and visualization performance of various methods including Ensemble on the DLPFC dataset, sample 151673.

**Figure 3:**
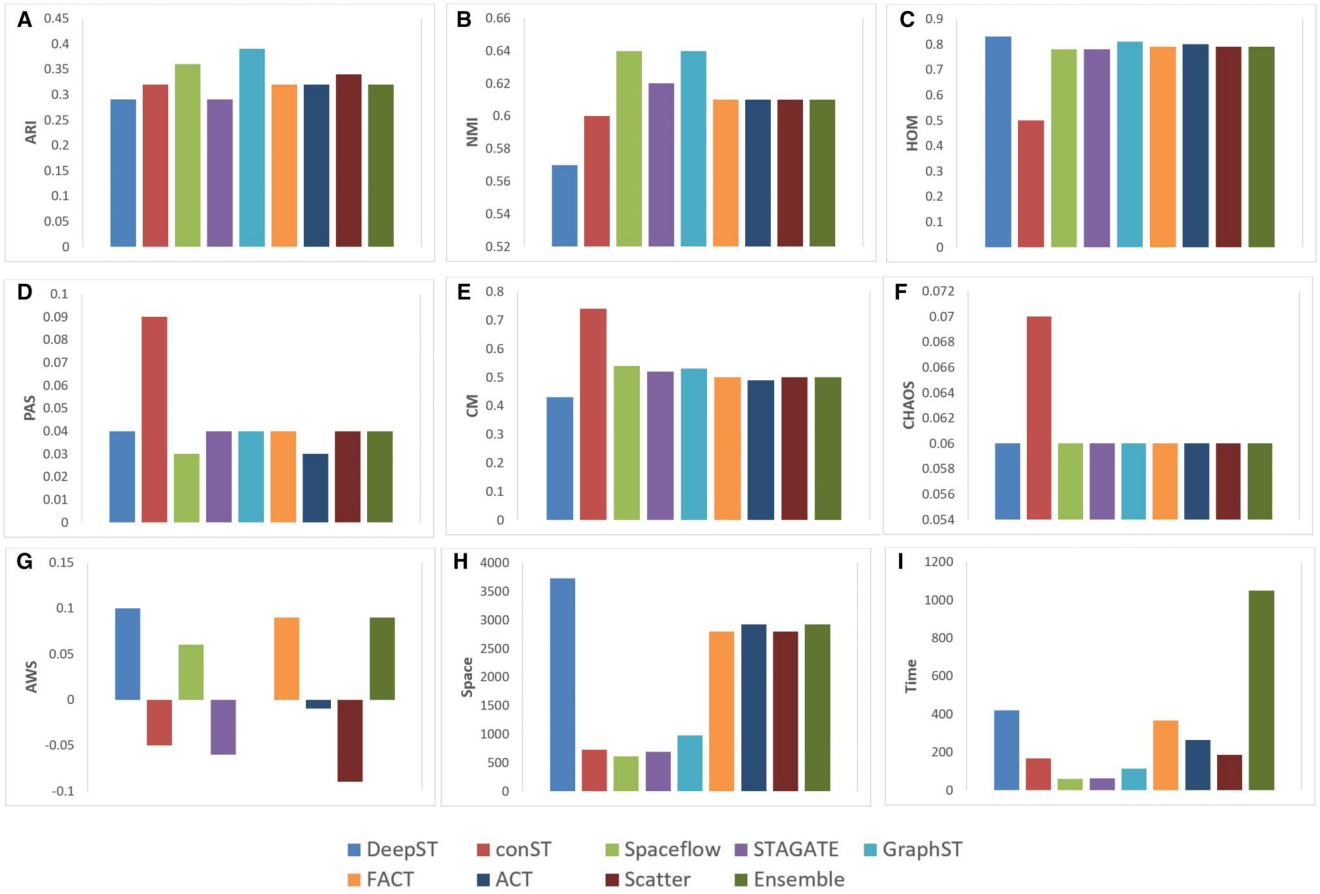
Comparison of spatial domain identification performance of various methods including the proposed method on the mouse brain anterior dataset.

Table 4 summarizes the ARI performance of competing methods across 12 DLPFC slides. Overall, the proposed ensemble variants provide the strongest and most robust performance: **Ensemble (ASW)** achieves the highest average ARI (0.517±0.091), while **Ensemble** attains 0.512±0.082 with a median of 0.500, indicating consistently strong performance across heterogeneous tissue sections. In terms of per-slide wins (best or tied-best ARI), **Ensemble** is top-performing on 6/12 slides (151507, 151508, 151510, 151670, 151673, and 151675), demonstrating reliable generalization across samples. Among individual baselines, GraphST and STAGATE remain competitive (medians 0.485 and 0.510, respectively), and achieve the best ARI on several slides (GraphST: 4/12; STAGATE: 3/12), while FACT is also strong (3/12) and attains the best ARI on slides 151509 and 151671. Notably, Scatter achieves the highest ARI on slide 151672 (0.734), suggesting that gene filtration can be particularly beneficial for slides with strong expression-dominant signals, whereas ACT yields the best ARI on slide 151674 (0.580), highlighting the value of joint representation–clustering optimization in certain tissue contexts. In contrast, SpaceFlow exhibits substantially lower performance overall (median 0.260), indicating limited effectiveness on this dataset. Taken together, these results show that (i) Scatter/ACT/FACT each capture complementary strengths on different slides, and (ii) the ensemble strategy leverages this complementarity to improve overall robustness and average performance relative to single-method baselines. Overall, these results highlight the stable and superior performance of our approach compared to current state-of-the-art methods. Comparisons of ARI scores across different variants of the proposed framework and against state-of-the-art methods on the DLPFC datasets are shown in Figs 2 and 3 (Supplementary Document 1), available as supplementary data at *Bioinformatics Advances* online.

**Table 4:** Performance comparison across different methods and samples (best scores in bold).^a^

Method	151507	151508	151509	151510	151669	151670	151671	151672	151673	151674	151675	151676	**Mean** ± **SD**
conST	0.260	0.370	0.420	0.330	0.300	0.230	0.380	0.380	0.510	0.450	0.440	0.390	0.372±0.081
DeepST	0.530	0.440	0.490	0.460	0.330	0.330	0.490	0.500	0.590	0.430	0.520	0.490	0.467±0.076
SpaceFlow	0.310	0.260	0.210	0.210	0.200	0.170	0.260	0.280	0.350	0.340	0.250	0.290	0.261±0.056
STAGATE	**0.590**	0.430	0.430	0.430	0.390	**0.510**	0.300	0.540	0.510	0.540	**0.570**	0.510	0.479±0.084
SpaGCN	0.430	0.380	0.370	0.400	0.300	0.210	0.380	0.510	0.480	0.410	0.390	0.320	0.382±0.080
GraphST	0.430	**0.490**	0.420	**0.510**	**0.430**	0.380	0.600	0.610	0.630	0.430	0.480	**0.570**	0.498±0.085
Scatter	0.471	0.397	0.481	0.384	0.395	0.377	0.468	**0.734**	0.661	0.546	0.457	0.425	0.483±0.113
ACT	0.486	0.336	0.484	0.350	0.372	0.386	0.621	0.582	0.661	**0.580**	0.322	0.369	0.462±0.122
FACT	0.554	0.313	**0.514**	0.441	0.392	0.407	**0.635**	0.605	**0.668**	0.467	0.253	0.259	0.459±0.141
Ensemble (ASW)	**0.590**	**0.490**	0.430	0.441	0.395	0.407	**0.635**	0.582	0.661	**0.580**	0.480	0.510	0.517±0.091
Ensemble (PAS)	0.554	**0.490**	**0.514**	**0.510**	0.395	0.407	0.621	0.582	0.661	0.467	0.457	0.369	0.502±0.091
Ensemble (CHAOS)	0.554	0.336	0.430	0.350	0.395	**0.510**	0.621	0.582	0.630	**0.580**	0.480	0.510	0.498±0.102
Ensemble	**0.590**	**0.490**	0.481	**0.510**	0.395	**0.510**	0.468	0.605	**0.668**	0.430	**0.570**	0.425	0.512±0.082

a Mean ± SD is computed across the 12 slides.

NMI, ARI, Geary’s *C*, Moran’s *I*, ASW, PAS, CHAOS, Completeness, and Homogeneity scores, along with execution time and memory usage for the DLPFC datasets, are provided in Supplementary Equation section of Supplementary Document 1, available as supplementary data at *Bioinformatics Advances* online. The mean runtimes on the DLPFC dataset were 39.93 s for SpaceFlow, 53.01 s for STAGATE, 66.18 s for GraphST, 183.24 s for Scatter, 312.64 s for ACT, 422.90 s for FACT, 579.52 s for DeepST, and 621.58 s per slide for Ensemble. These results show that our framework is not the fastest in absolute runtime; rather, its contribution is a stronger robustness–accuracy tradeoff, with the Ensemble variant achieving the best overall mean ARI and the lowest variance among the proposed variants.

Figure 2 provides the clustering visualization obtained from different methods applied to the 151673 DLPFC sample. Each spot on the H&E image of the sample is color-coded according to the cluster assignment determined by the respective methods. Our method achieves the highest performance with an ARI of 0.67. This score closely aligns with the ground-truth annotations. The resulting clusters exhibit well-defined and contiguous cortical boundaries, effectively preserve the spatial continuity of cortical layers compared to other methods, proposed framework more distinctly separates adjacent layers and reduces boundary mixing. The cluster visualization supports the quantitative advantage indicated by the ARI, demonstrating the consistent accuracy of the Ensemble method in recovering spatial domains that align with anatomical structures. Figure 1 (Supplementary Document 1), available as supplementary data at *Bioinformatics Advances* online summarizes the comparative performance of the proposed framework and baseline methods on the DLPFC dataset. Well-defined boundaries and alignment with known cortical layers demonstrate its ability to capture biologically relevant structures, underscoring the robustness of the method. The number of expected clusters for clustering algorithms after embedding generation was set to 7 for all algorithms. However, from embeddings, only six clusters were extracted reliably for ACT, FACT, and Ensemble. Still, the ARI values were acceptable. The six-cluster solution produced the most stable and spatially coherent laminar partition without introducing unnecessary fragmentation for the said methods.

#### 3.2.1 Qualitative failure cases and biological interpretation

**Figure 4:**
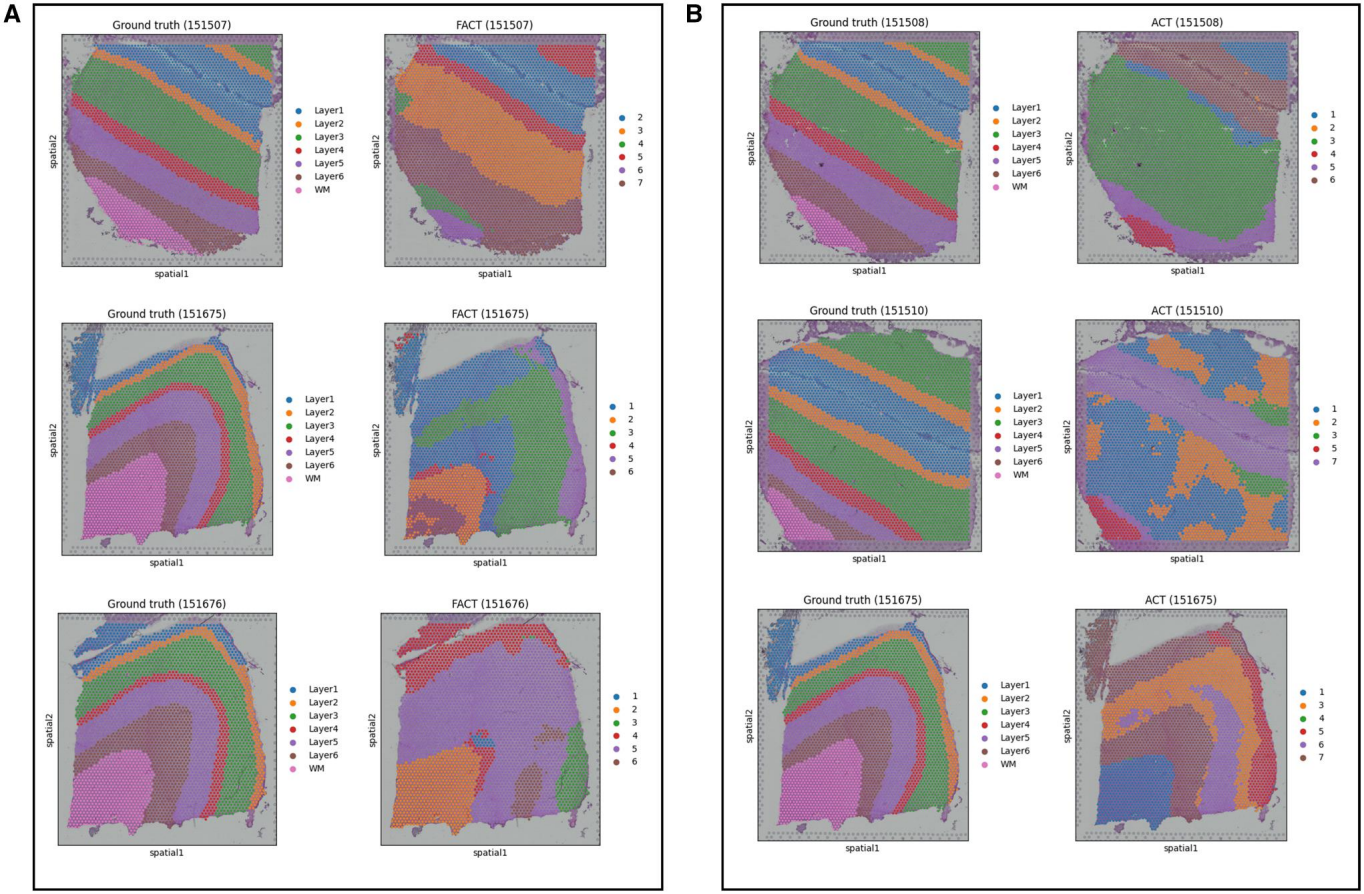
Representative failure cases for ACT/FACT on DLPFC. Selected tissue sections where ACT or FACT deviates from the laminar ground-truth organization. (A) ACT failures (e.g. 151508, 151510, 151675) are characterized by either over-smoothing/under-segmentation where multiple cortical layers are merged into a few dominant regions, or patchy discontinuities that break laminar continuity. (B) FACT failures (e.g. 151675, 151676) often manifest as layer collapsing into broad domains and boundary drift, suggesting reduced separability of adjacent laminae after filtration.

Figure 4 illustrates representative DLPFC sections where ACT or FACT performs worse, despite strong overall quantitative results. ACT learns a latent representation and optimizes it jointly with a clustering objective, followed by spatially informed label refinement (using spatial neighborhoods) that encourages local consistency. While this refinement improves coherence in many slices, it can over-smooth thin or weakly separated laminae: narrow cortical bands may be absorbed into the dominant label of nearby regions, leading to layer merging and reduced ARI. This behavior is visually apparent in slide 151508, where several diagonal laminar stripes in the ground truth are replaced by a few large contiguous regions in ACT.

ACT can also fail when early cluster assignments are unstable due to low signal-to-noise, local dropout, or strong expression overlap between neighboring layers. Because ACT performs iterative self-training to align embedding structure with its current cluster assignments, early mistakes may be amplified rather than corrected, producing patchy or mixed domains that violate laminar continuity, as observed in slides 151510 and 151675.

FACT inherits ACT’s spatial refinement but additionally integrates a gene filtration strategy (Scatter-style filtration) intended to remove highly prevalent genes and emphasize more discriminative signals. Although beneficial in many cases, this filtration can be detrimental in certain DLPFC sections where laminar identity is encoded by broad gene programs with subtle gradients rather than by sparse markers alone. In such slices, removing globally prevalent genes can suppress weak-but-consistent laminar signals and reduce separability between adjacent layers, yielding collapsed domains and boundary drift. This effect is particularly evident in highly curved cortical sections (e.g. 151675 and 151676), where layer thickness varies spatially and boundaries are strongly non-linear.

These qualitative failures are also biologically plausible given cortical organization and the Visium measurement process. DLPFC laminar boundaries are not perfectly discrete: layers contain partially overlapping mixtures of cell types, and transitions can be gradual (especially near gyral curvature and white-matter interfaces). Moreover, each Visium spot aggregates multiple cells (partial-volume mixing), so spots along boundaries naturally exhibit hybrid expression. Under these conditions, a strong spatial coherence prior can over-regularize true thin layers (merging), while aggressive filtration can remove subtle gradient signals (collapsing), explaining the slice-dependent degradation observed in Fig. 4. These slice-specific failure modes motivate the use of an ensemble that leverages complementary strengths across methods rather than relying on a single variant for all tissue sections.

#### 3.2.2 Sensitivity of scatter to the knob parameter

In addition to the knob =100 Scatter results reported in Table 3, we evaluated knob∈{80,120} on the same 12 DLPFC slides and compared performance using ARI. Overall, knob=80 yields the best average agreement with cortical-layer annotations (mean ARI =0.525±0.099 across 12 slides), outperforming knob =120 (mean ARI =0.493±0.067) and improving over the knob =100 baseline in Table 3 (mean ARI ≈0.484±0.111). The improvements with knob =80 are particularly pronounced on difficult tissue sections where the baseline configuration under-segments laminar structure: for example, slide 151508 increases from 0.40 (knob =100) to 0.571 (knob =80), and slide 151671 increases from 0.47 to 0.612. Importantly, knob =80 also preserves strong performance on the best-performing section 151672 (ARI 0.734), which remains comparable to knob =100.

knob =120 produces slightly more conservative and less variable performance (smaller standard deviation, 0.067), and it achieves the best ARI on a subset of slides (e.g. 151509: 0.504; 151510: 0.501; 151674: 0.554; 151676: 0.430). However, knob =120 can reduce agreement on slides where fine-grained laminar boundaries are important, most notably 151672 (0.599 versus 0.73 at knob =100) and 151673 (0.511 versus 0.66 at knob =100), suggesting that larger knob values may over-smooth or merge subtle layer transitions in some sections. Taken together, these results indicate that Scatter remains competitive across a broad knob range (80–120), with knob =80 offering the best mean ARI (higher accuracy on average) and knob =120 offering increased stability (lower variance) at the cost of reduced performance on specific high-signal laminar sections.

### 3.3 Performance evaluation on Visium 10x mouse brain anterior dataset

To further evaluate the model’s performance, it was applied to the mouse brain anterior ST dataset (10x Genomics 2020a). Gene expression across the anterior mouse brain is profiled in this dataset while critical spatial context is preserved. This dataset is widely used to investigate brain structure, function, and spatially organized cell types, enabling the identification of distinct spatial domains, region-specific gene activity, and architectural features. The original dataset contains 52 clusters; for comparative purposes, this was streamlined to 12 clusters to facilitate interpretation and enhance comparability.

**Figure 5:**
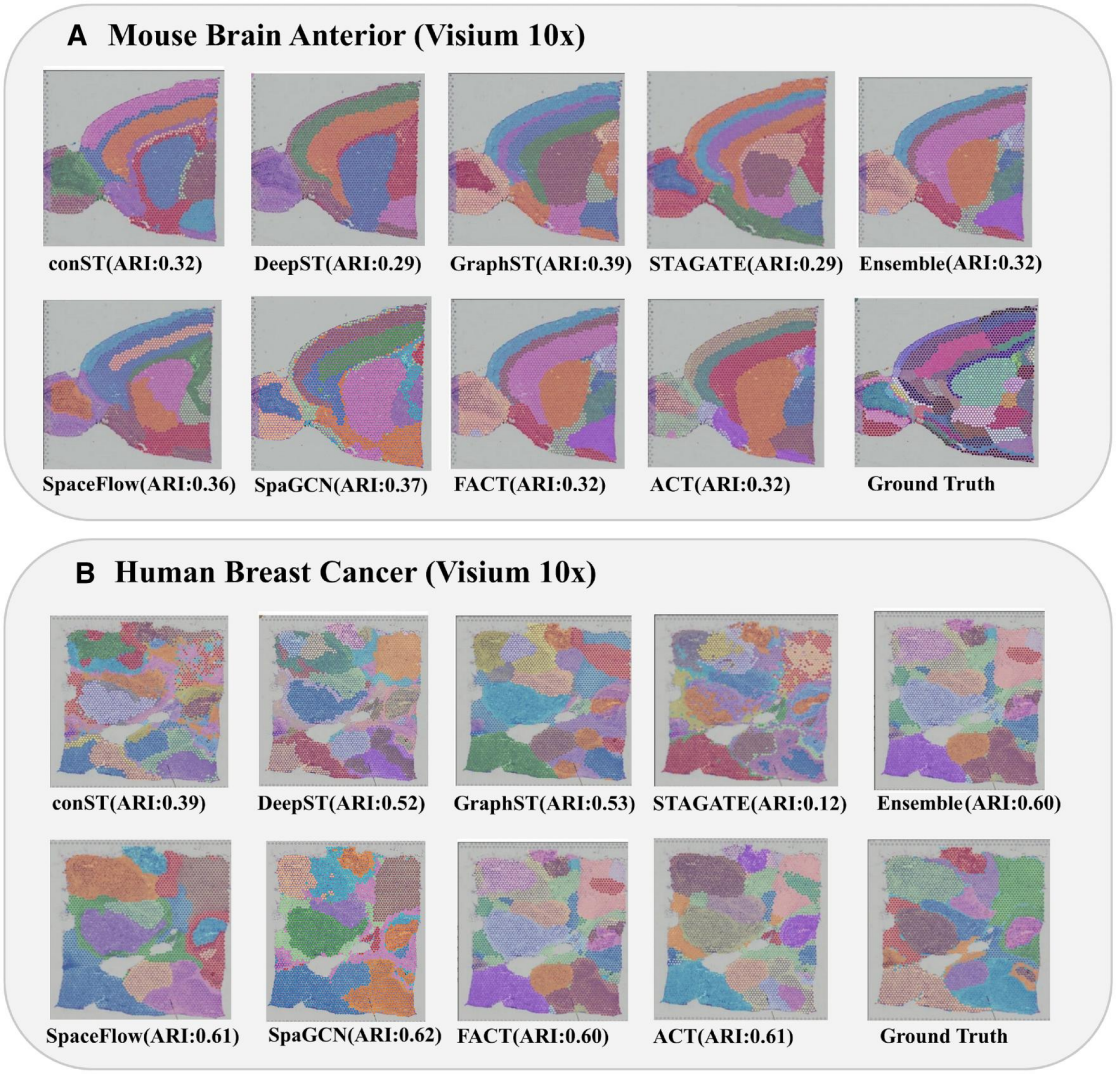
Visual comparison of clustering performance across state-of-the-art methods and the proposed approach against the ground truth. (A) Mouse brain anterior dataset. (B) human breast cancer dataset.

As illustrated in Fig. 5A, the clustering performance on the mouse brain anterior Visium 10x dataset align more closely with ground-truth annotations compared to those produced by other state-of-the-art methods. Robust and competitive performance is also observed across diverse spatial clustering metrics. Although GraphST recorded the highest ARI at 0.39, our models excelled in NMI, achieving a commendable 0.61, alongside notable spatial homogeneity (HOM≥0.79). As shown in Fig. 3, the ACT and FACT variants achieved an ARI of 0.32 but did not surpass the performance of GraphST or SpaceFlow. In contrast, the other variant Scatter (ARI: 0.34) consistently outperformed most of the state-of-the-art methods on the mouse brain anterior dataset, demonstrating superior clustering accuracy and efficiency across multiple evaluation metrics.

#### 3.3.1 Fine-grained mouse brain anterior result analysis

A detailed analysis of the mouse brain anterior dataset was conducted to identify all 52 clusters. The mouse brain anterior Visium section exhibits multiple distinct anatomical motifs, providing a valuable framework for assessing spatial clustering.

**Figure 6:**
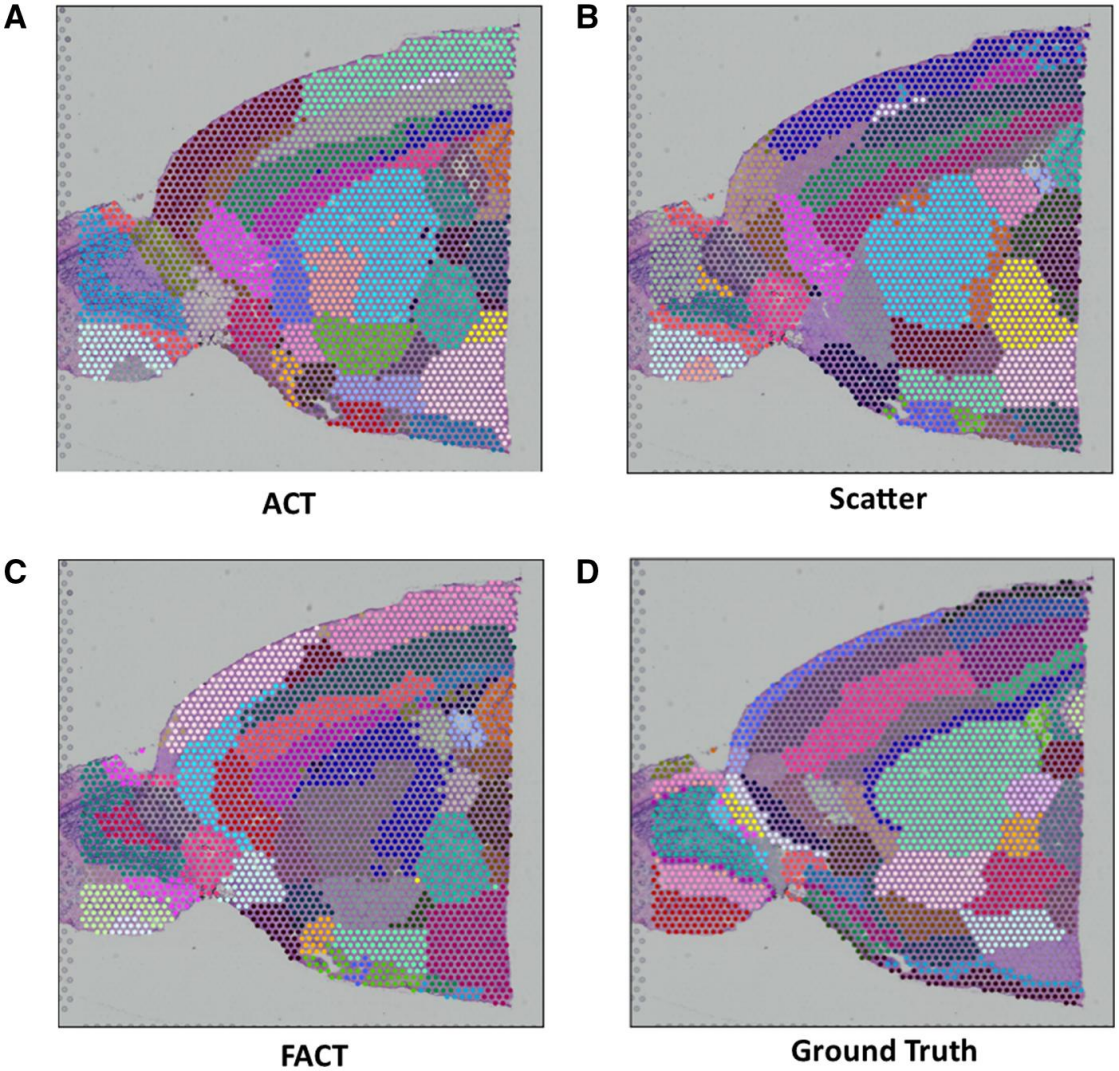
Visual comparison of clustering performance across proposed approaches for 52 clusters of mouse brain anterior dataset.

ACT provides the most biologically convincing overall reconstruction (Fig. 6A). ACT preserves the separation between the left olfactory compartment and the main cerebrum, reconstructs curved dorsal bands that are consistent with cortical lamination, and at the same time keeps the large central deep region compact while still subdividing the ventral forebrain into several coherent domains. This is biologically important because the anterior mouse brain is not purely layered tissue: the dorsal cortex is laminar, but the ventral and deep forebrain are organized as neighboring nuclei and compartments. ACT captures this dual organization better than the other methods. Overall, ACT best preserves the hierarchical structure of the tissue by recovering both cortex-like ribbon organization and nucleus-like subcortical organization.

FACT’s key visual feature is the dorsal cortical pattern, with smooth, laminar bands capturing broad spatial gradients (Fig. 6C). However, deeper and ventral regions are over-merged, reducing subcortical specificity and collapsing some ventral and lateral territories. Overall, FACT emphasizes laminar cortical organization at the expense of finer deep-forebrain heterogeneity.

Meanwhile, Scatter delivers the cleanest coarse partition, separating the olfactory region, producing a coherent central deep domain, and forming smooth, compact regions with minimal fragmentation (Fig. 6B), making it biologically plausible at the macro-anatomical level. Its main limitation is under-resolution: dorsal cortical areas appear as broad ribbons, and several ventral compartments merge into larger domains. Thus, Scatter is effective for coarse atlas-like segmentation but less capable of capturing fine-grained anatomy compared with ACT.

The ground truth (Fig. 6D) comprises both layered cortex and heterogeneous basal forebrain, favoring approaches that balance these two anatomical principles. ACT emerges as the most effective, preserving dorsal cortical ribbons while maintaining coherent and distinct subcortical and ventral domains. Scatter performs moderately well, capturing the broad anatomical structure smoothly but tending to merge finer boundaries. FACT, in contrast, overemphasizes cortical lamination at the expense of deep and ventral specificity.

These results suggest that the methods operate at different scales: FACT highlights cortical laminar organization, Scatter provides smooth coarse regional partitioning, and ACT delivers the most anatomically faithful representation across the entire anterior mouse brain section.

Table 5 summarizes the performance of ACT, Scatter, and FACT on the 52-cluster mouse brain anterior dataset, highlighting their relative strengths in cortical and subcortical organization.

**Table 5:** Performance comparison for 52-cluster mouse brain anterior dataset.

Method	ARI	NMI	Performance summary
ACT	0.4579	0.7149	**Cortical ribbons**: strong (curved, laminar bands); **Subcortical specificity**: strong (distinct ventral & deep domains); **Overall fidelity**: highest; Strengths: balanced recovery of layered cortex and compact nuclei; **Limitation**: mild over-smoothing in thin dorsal regions
Scatter	0.4242	0.7052	**Cortical ribbons**: moderate (broad ribbons); **Subcortical specificity**: moderate (coarse compartment separation); **Overall fidelity**: high (coarse scale); **Strengths**: clean and spatially coherent macro-structure; **Limitation**: under-resolution of fine anatomical details
FACT	0.4099	0.7131	**Cortical ribbons**: very strong (smooth laminar ribbons); **Subcortical specificity**: weak (merged deep/ventral regions); **Overall fidelity**: moderate; **Strengths**: excellent cortical gradient capture; **Limitation**: over-merging of subcortical territories

Evaluation at the full 52-cluster resolution further improves both performance and biological relevance, better demonstrating the advantages of the proposed approach; detailed results are provided in the Supplementary Material, available as supplementary data at *Bioinformatics Advances* online (Section 3.4).

Overall, ACT best captures the tissue’s mixed anatomy, preserving layered dorsal cortex alongside compact, heterogeneous deep and ventral forebrain compartments, whereas Scatter excels at coarse-scale structure and FACT overemphasizes laminar organization at the expense of subcortical specificity.

### 3.4 Performance evaluation on Visium 10x breast cancer spatial profiles

**Figure 7:**
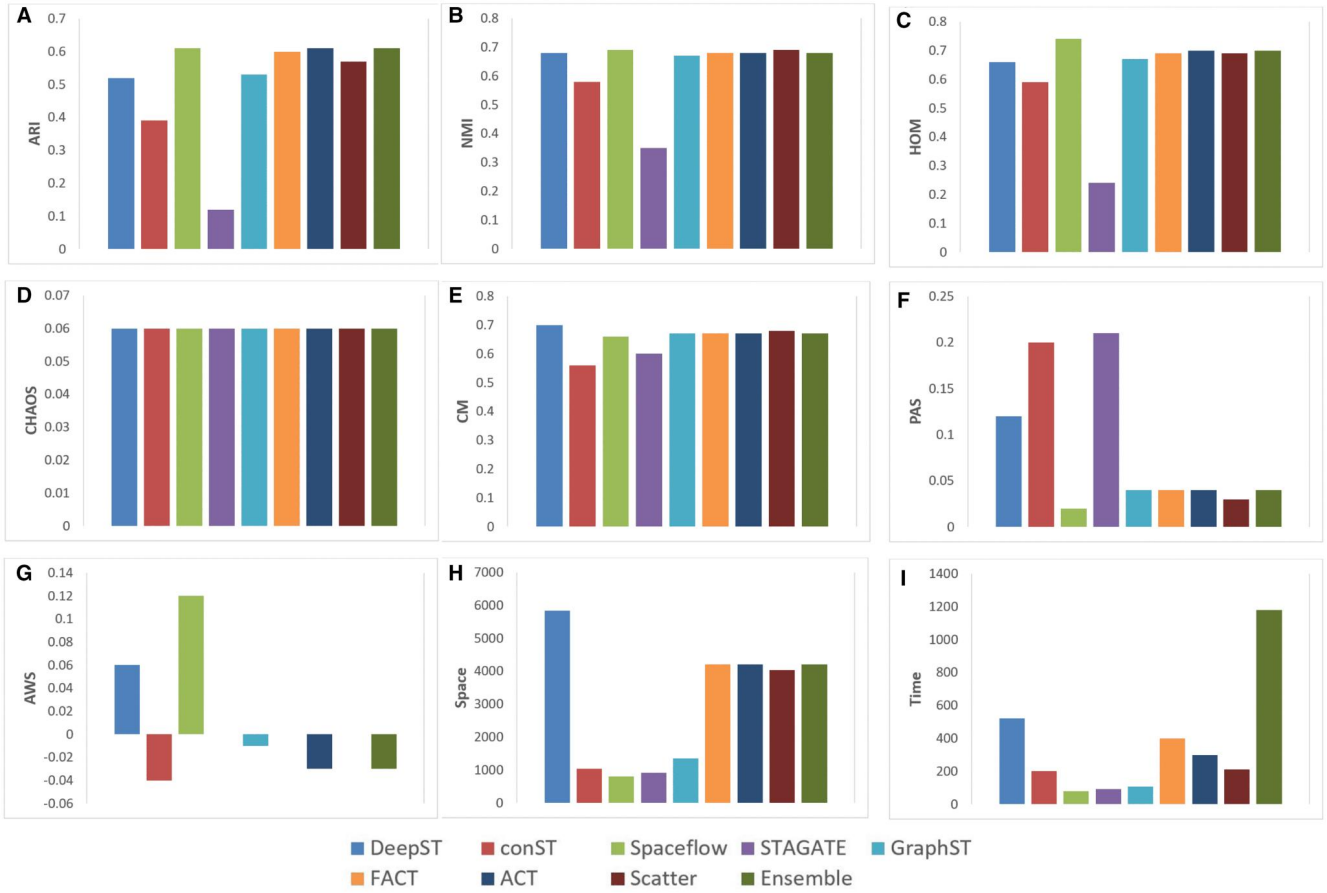
Clustering performance of proposed method and other state-of-the-art methods on human breast cancer dataset using different evaluation matrices.

To ensure robustness, generalization, and unbiased performance, the proposed method was evaluated on publicly available human breast cancer spatial dataset (10x Genomics 2020b). This dataset provides spatially-resolved transcriptomic profiles across histologically annotated breast cancer tissue, comprising 3798 high-quality spots and more than 18 000 genes. The number of clusters applied for the analysis of clustering performance is 12. The ACT and Ensemble methods achieved the competitive ARI values, demonstrating the effectiveness of our custom approach. As shown in Fig. 7, the proposed framework achieves competitive performance to state-of-the-art methods on the human breast cancer dataset across a comprehensive set of clustering evaluation metrics.

Figure 5B shows the clustering visualization performance on the human breast cancer Visium 10x dataset. The Ensemble method and FACT achieve high ARI scores of 0.6, respectively—outperforming DeepST (0.52), GraphST (0.53), conST (0.39), and STAGATE (0.12). Also, ACT, with high ARI, demonstrates strong alignment with the ground truth and consistent spatial coherence across both tumor and microenvironment regions. Both FACT and ACT effectively delineate biologically meaningful domains, underscoring their strength in capturing the complex spatial organization of breast cancer tissue. The results closely match the manually annotated areas. The identified domains are more fluent and continuous than those from other spatial algorithms, reflecting an improved ability to resolve complex tissue structures.

### 3.5 Overall comparison on 10x Visium datasets

Within the DLPFC dataset, our proposed framework secures the highest performance in six out of 12 slides. In contrast, STAGATE achieves the leading result in four slides, while GraphST takes the top position in two slides. The remaining methods, conST, DeepST, SpaceFlow, and SpaGCN, do not outperform our framework on any of the DLPFC slides. Table 6 presents the comparative performance of the proposed framework against state-of-the-art methods in the DLPFC, mouse brain anterior, and human breast cancer datasets.

**Table 6:** Overall performance comparison of different methods across DLPFC, mouse brain, and human breast cancer datasets based on ARI.

Method	DLPFC (12 slides)	Mouse brain anterior	Human breast cancer
conST			
DeepST			
SpaceFlow		Ranked third	Ranked second
STAGATE	Outperformed in four slides		
SpaGCN		Ranked second	Ranked first
GraphST	Outperformed in two slides	Ranked first	
Proposed framework	Outperformed in six slides		Ranked third

In the human breast cancer dataset, both our frameworks reach a high rank. Yet, unlike in DLPFC—where STAGATE and GraphST show strong outcomes in four and two slides, respectively—both approaches perform substantially weaker here. Our framework achieves an ARI of 0.61, whereas STAGATE and GraphST achieve only 0.12 and 0.53, respectively.

For the Mouse Brain dataset, SpaceFlow (0.36) and GraphST (0.39) show slightly higher scores than our framework (0.34). Even so, SpaceFlow does not surpass in any of the DLPFC slides, and GraphST relatively underperformed in the Breast Cancer dataset.

### 3.6 Performance on Slide-seqV2 (mouse hippocampal formation)

**Figure 8:**
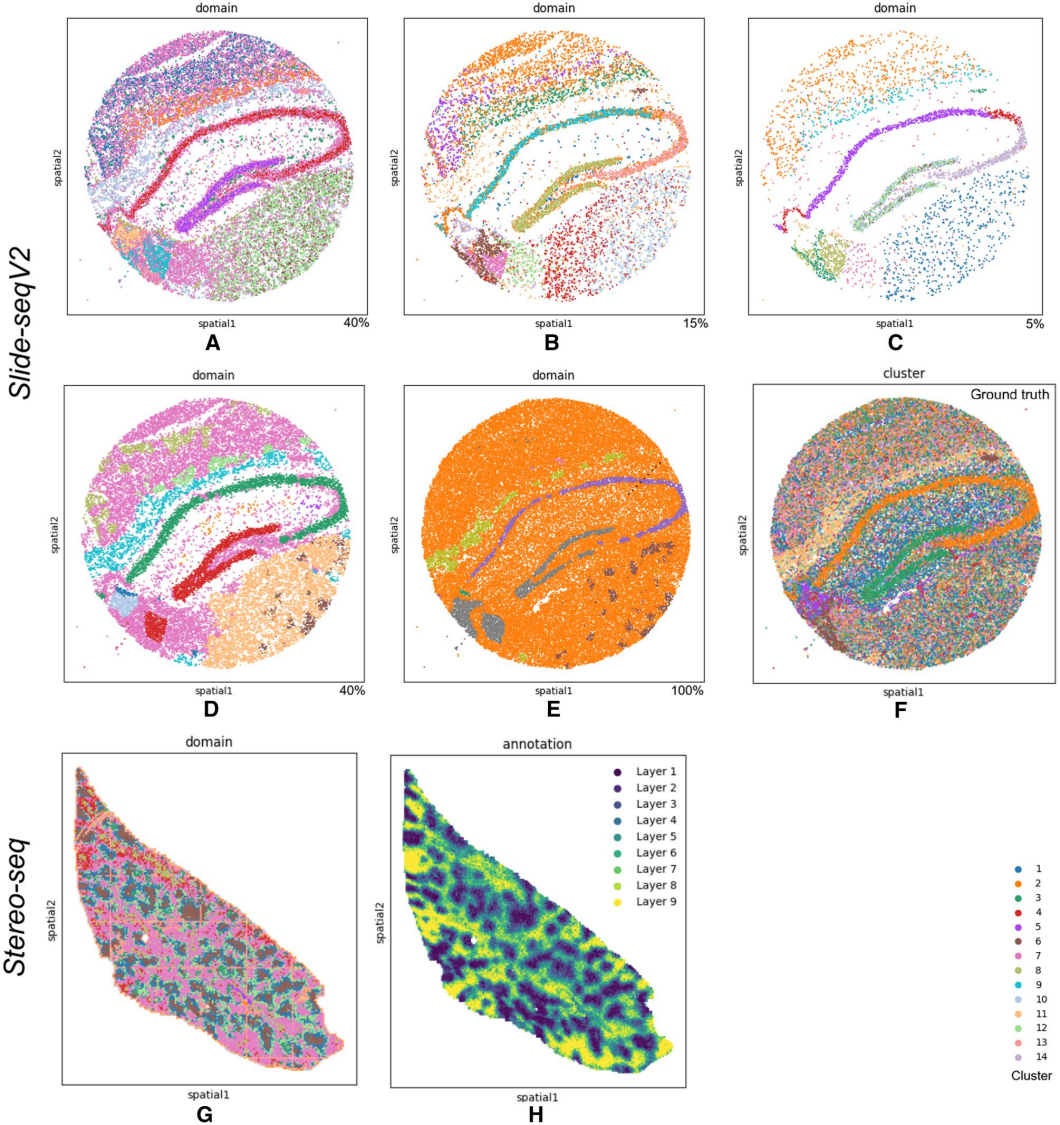
ACT on Slide-seqV2 hippocampal formation and Stereo-seq liver. *Slide-seqV2*: (A–C) ACT-inferred domains using the top 40%, 15%, and 5% of selected locations (with refinement); (D) top 40% without refinement; (E) all locations (100%); (F) reference labels/annotation. *Stereo-seq*: (G) ACT-inferred domains; (H) reference layer annotation (Layer 1–Layer 9).

In the Slide-seqV2 hippocampal formation section, ACT recovers spatial domains that are consistent with hippocampal cytoarchitecture, including prominent curved/laminar structures and adjacent subfield-scale compartments (Rodriques *et al.* 2019, Stickels *et al.*, 2021). Notably, the dominant hippocampal geometry is preserved under substantial changes in the location-selection percentage: the characteristic arc-like bands remain continuous and readily identifiable when using 40%, 15%, and 5% of selected locations (Fig. 8A–C), indicating robustness to sampling density and parameter variation. Spatial refinement further improves anatomical plausibility: at matched selection (40%), the refined solution (Fig. 8A) yields smoother, more contiguous regions than the non-refined counterpart (Fig. 8D), consistent with refinement reducing isolated label fluctuations. When all locations are included, the segmentation is dominated by a broad background-like region (Fig. 8E), suggesting that prioritizing informative locations can enhance anatomical contrast in bead-based assays. Despite the fine-grained and spatially mixed reference labels (Fig. 8F), ACT consistently preserves the major curved structures that define hippocampal organization (Fig. 8A–C), supporting its use for anatomically interpretable domain discovery.

**Figure 9:**
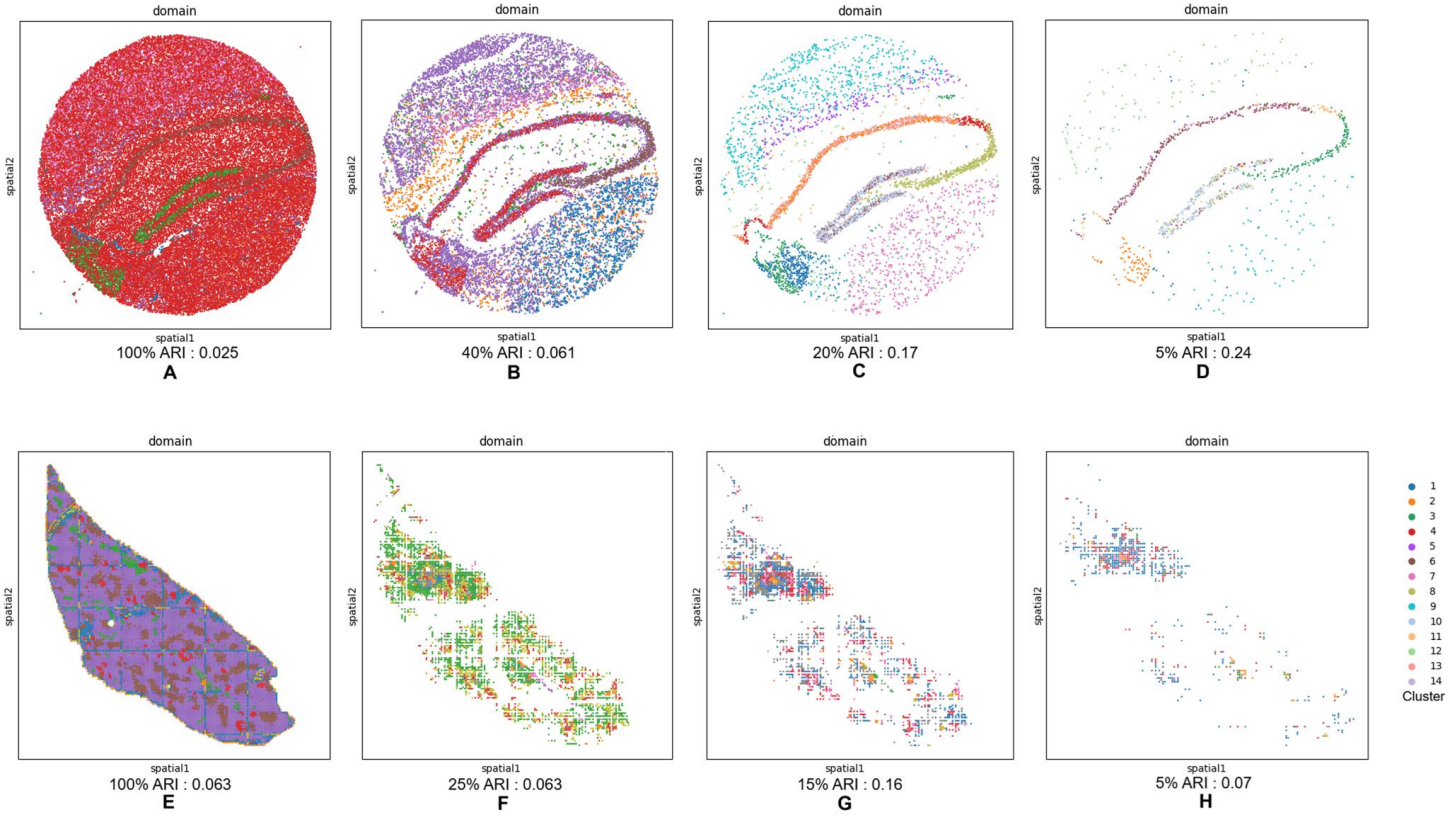
FACT on Slide-seqV2 hippocampal formation and Stereo-seq liver across location-selection percentages. *Slide-seqV2*: (A) 100% (ARI =0.025); (B) 40% (ARI =0.061); (C) 20% (ARI =0.17); (D) 5% (ARI =0.24). *Stereo-seq*: (E) 100% (ARI =0.063); (F) 25% (ARI =0.063); (G) 15% (ARI =0.16); (H) 5% (ARI =0.07).

FACT exhibits a clear improvement as location selection becomes more stringent on Slide-seqV2 (Fig. 9A–D). With 100% of locations, the result is dominated by a single large domain and agreement with the reference is low (ARI=0.025; Fig. 9A), consistent with low-information/background locations obscuring laminar boundaries. At 40% selection, hippocampal arc-like structure becomes visible and agreement increases (ARI=0.061; Fig. 9B). Further selection strengthens anatomical delineation: 20% yields crisp laminar arcs and compartments (ARI=0.17; Fig. 9C), and even at 5% the dominant hippocampal geometry remains evident with the highest agreement (ARI=0.24; Fig. 9D). Biologically, this pattern is consistent with the hippocampus containing strong, spatially autocorrelated transcriptional programs, such that retaining the highest-signal locations is sufficient to recover laminar structure while reducing noise that blurs boundaries.

**Figure 10:**
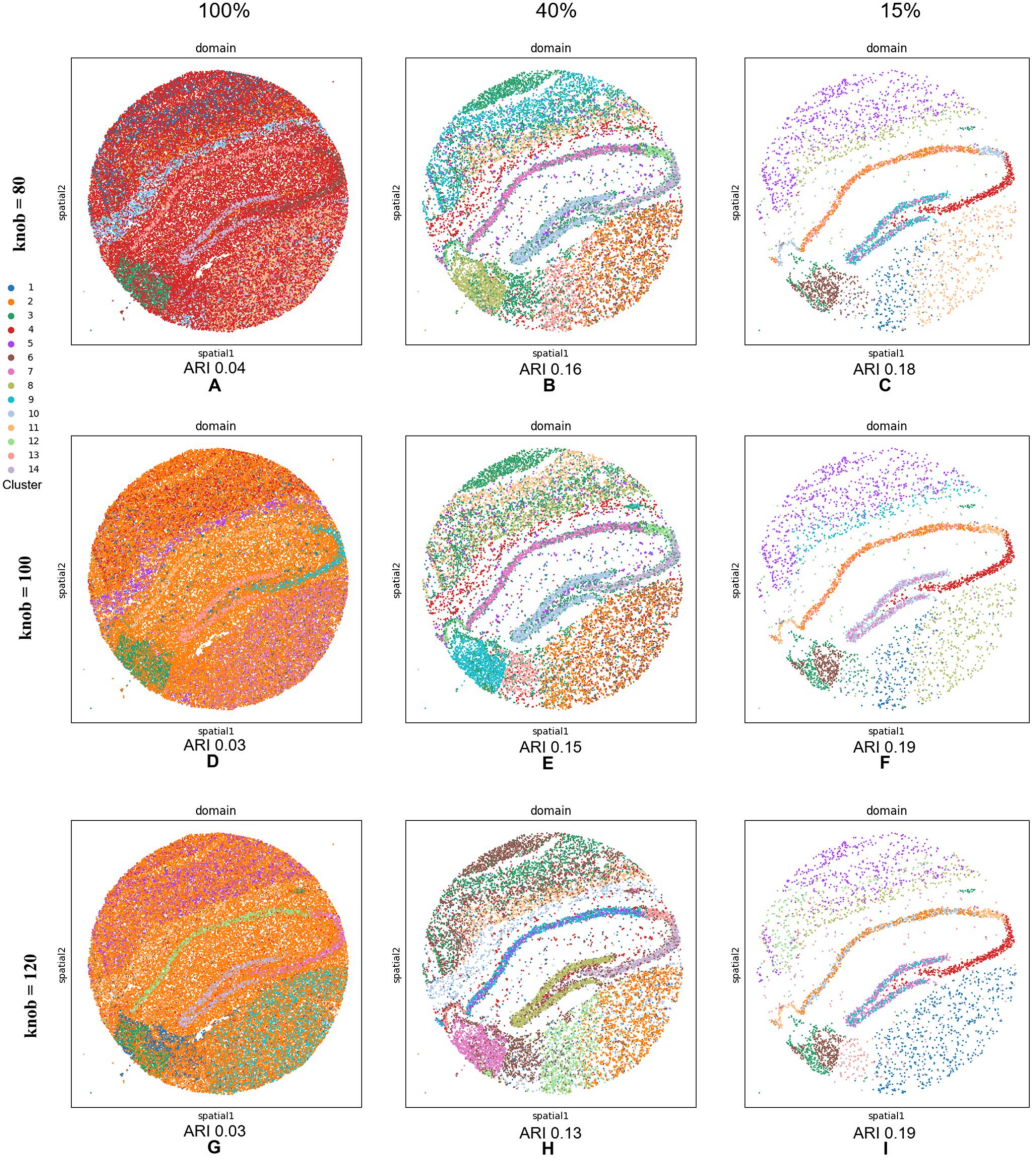
(A-I) Scatter on Slide-seqV2 across location selection and parameter *knob*. Columns vary the selected-location percentage (100%, 40%, 15%), and rows vary *knob* (knob=80,100,120). ARI values are reported in each panel.

Across the Scatter parameter grid (rows: *knob*; columns: selected-location percentage), Slide-seqV2 results show strong sensitivity to location selection but relative stability across *knob* once selection is applied (Fig. 10A–I). Using all locations yields consistently low agreement and broad, less anatomically specific segmentation across tested *knob* values (ARI=0.04,0.03,0.03; Fig. 10A, D, G). In contrast, moderate selection sharply improves both agreement and anatomical interpretability: at 40% selection, curved hippocampal bands and major compartments emerge robustly across *knob* (ARI=0.16,0.15,0.13; Fig. 10B, E, and H). At 15% selection, agreement increases further (ARI=0.18,0.19,0.19; Fig. 10C, F, and I) while preserving the same hallmark hippocampal geometry. Variation in *knob* primarily modulates granularity (boundary smoothness versus finer subdivision) rather than altering the core laminar pattern, indicating that Scatter can reliably recover the dominant hippocampal domains without requiring narrow hyperparameter tuning.

### 3.7 Performance on Stereo-seq (mouse liver; lobule zonation)

In the Stereo-seq liver sample, ACT produces spatially coherent domains that tile the tissue section (Fig. 8G) in a manner consistent with the presence of sub-lobular functional organization and repeating lobular territories. Liver biology is characterized by spatially structured metabolic specialization (zonation) along the porto-central axis rather than a small set of globally homogeneous blocks (Halpern *et al.* 2017). Accordingly, ACT’s discrete domain map provides an analysis-ready partition, i.e. complementary to the continuous Layers 1–9 annotation (Fig. 8H): the layer map summarizes a smooth zonation axis, whereas ACT highlights discrete territories that can be used for region-level marker discovery, pathway enrichment, and detection of localized deviations from the global gradient (e.g. lobule-to-lobule heterogeneity or niche-like microterritories). Overall, ACT yields a structured segmentation that is biologically plausible for liver at Stereo-seq resolution and supports downstream, region-based interpretation.

On Stereo-seq liver, FACT shows the strongest performance at intermediate location selection (Fig. 9E–H). With 100% of locations, agreement with the layer annotation is modest (ARI=0.063; Fig. 9E), and selecting 25% does not substantially change performance (ARI=0.063; Fig. 9F). Selecting 15% yields a pronounced improvement (ARI=0.16; Fig. 9G), consistent with enhanced recovery of lobule-scale zonation structure once low-information/background locations are reduced. At very aggressive selection (5%), agreement decreases (ARI=0.07; Fig. 9H), plausibly reflecting insufficient spatial coverage to represent a continuous zonation axis. This behavior suggests that FACT benefits from denoising through selection, while retaining enough locations to preserve the spatial continuity of zonation.

**Figure 11:**
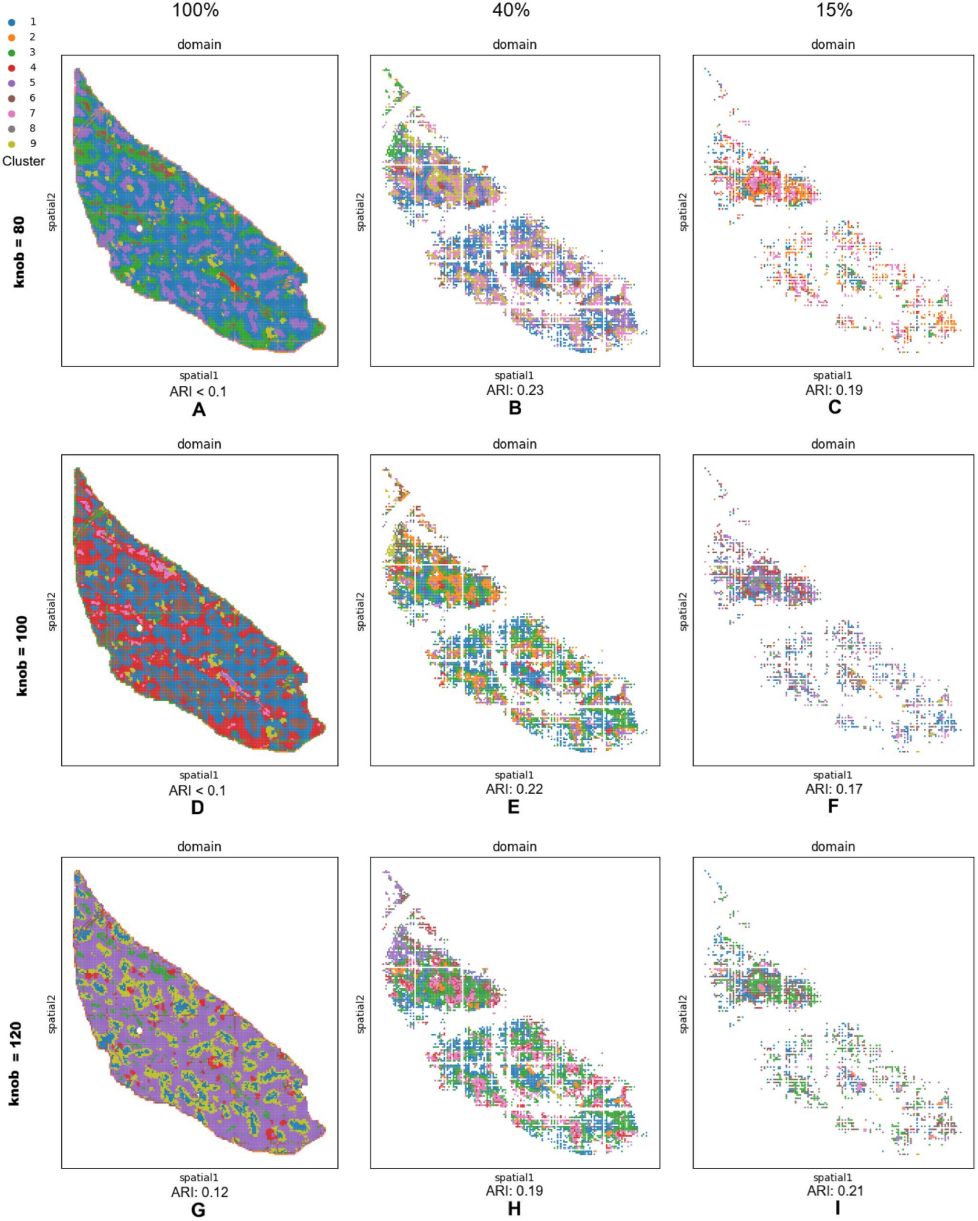
(A-I) Scatter on Stereo-seq liver across location selection and parameter *knob*. Columns vary the selected-location percentage (100%, 40%, 15%), and rows vary *knob* (knob=80,100,120). ARI values are reported in each panel.

Scatter further supports the above trends and demonstrates strong performance at moderate selection across a range of *knob* values (Fig. 11A–I). Using all locations yields low agreement for knob=80 and knob=100 (both ARI<0.1; Fig. 11A and D), improving only slightly at knob=120 (ARI=0.12; Fig. 11G), consistent with ultra-high-resolution heterogeneity and technical variability obscuring zonation when all locations are included. Selecting 40% of locations produces the highest agreement across the grid (ARI=0.23,0.22,0.19; Fig. 11B, E, and H), and selecting 15% retains strong agreement (ARI=0.19,0.17,0.21; Fig. 11C, F, and I). The consistency of these results across *knob* indicates that Scatter is capturing a reproducible zonation-related spatial signal rather than a parameter-specific artifact, while providing flexibility to adjust domain granularity via *knob* without disrupting correspondence to the layer annotation.

### 3.8 Statistical significance analysis

To quantify whether performance differences in Table 4 are statistically significant across multiple DLPFC slides, we performed non-parametric repeated-measures testing on ARI values (12 paired slides). We first applied a Friedman test over all compared methods, which detects overall differences in central tendency without assuming normality. The Friedman test was significant ($\chi^{2}=55.26$, *P* = 1.09 × 10^−8^), indicating that at least one method differs from the others.

We then performed *post hoc* paired Wilcoxon signed-rank tests (one-sided; $H_{0}:{\text{ARI}}_{A}\leq {\text{ARI}}_{B}$ and $H_{1}:{\text{ARI}}_{A}>{\text{ARI}}_{B}$) to evaluate whether the proposed methods (Scatter, ACT, FACT, and Ensemble) outperform representative baselines (conST, DeepST, SpaceFlow, SpaGCN) as shown in Table 7. For each proposed method, we corrected the four baseline comparisons using Holm’s procedure. As an effect size, we report the rank-biserial correlation ($r_{\text{rb}}$), where larger positive values indicate stronger, more consistent improvements.

**Table 7:** Post-hoc paired Wilcoxon signed-rank tests across 12 slides (one-sided; Holm-corrected per proposed method over four baselines).^a^

Proposed	Baseline	Wins	Δ **Mean**	$p_{\text{Holm}}$	$r_{\text{rb}}$
Scatter	conST	12/12	+0.111	$9.77\times 10^{-4}$ ***	1.000
	DeepST	5/12	+0.016	0.396	0.115
	SpaceFlow	12/12	+0.222	$9.77\times 10^{-4}$ ***	1.000
	SpaGCN	11/12	+0.101	$9.77\times 10^{-4}$ ***	0.974
ACT	conST	9/12	+0.091	0.0269 *	0.718
	DeepST	6/12	−0.004	0.545	−0.026
	SpaceFlow	12/12	+0.202	$9.77\times 10^{-4}$ ***	1.000
	SpaGCN	9/12	+0.081	0.0242 *	0.769
FACT	conST	9/12	+0.087	0.0959	0.564
	DeepST	8/12	−0.008	0.339	0.154
	SpaceFlow	11/12	+0.198	0.00293 **	0.949
	SpaGCN	9/12	+0.077	0.0959	0.615
Ensemble	conST	11/12	+0.140	$9.77\times 10^{-4}$ ***	0.974
	DeepST	8/12	+0.045	0.0226 *	0.682
	SpaceFlow	12/12	+0.251	$9.77\times 10^{-4}$ ***	1.000
	SpaGCN	12/12	+0.130	$9.77\times 10^{-4}$ ***	1.000

a “Wins” counts slides where the proposed method has higher ARI than the baseline. $r_{\text{rb}}$ is rank-biserial effect size.

* p<0.05 ,

** p<0.01 ,

*** p<0.001 .

In addition to improved averages, the Ensemble achieves the highest overall stability among the proposed variants (mean±std: Scatter 0.483±0.113, ACT 0.462±0.122, FACT 0.459±0.141, Ensemble 0.512±0.082). Moreover, when compared slide-by-slide against all methods in Table 4, Ensemble attains the best ARI in 6/12 slides (including ties), indicating robust performance under tissue heterogeneity.

#### 3.8.1 Rationale for improved performances

Scatter improves clustering by removing highly occurring genes, which reduces dominance of ubiquitous signals and increases discriminability of spatial domains. ACT further improves robustness by jointly optimizing latent representations with a clustering objective while incorporating spatial context, helping align learned embeddings with spatially coherent tissue structures. FACT combines Scatter-style filtration with the ACT optimization pipeline, enhancing feature selection before joint refinement.

Finally, Ensemble leverages complementarity among multiple strong candidates (ACT/FACT/Scatter/GraphST/STAGATE) and selects the output with the highest unsupervised cluster-quality score, which yields the best overall mean ARI and the smallest standard deviation across slides, consistent with increased robustness to slide-specific heterogeneity.

## 4 Discussion and conclusion

In this article, we propose a trainable, loss-optimization-based clustering framework comprising four configurable variants—Scatter, ACT, FACT, and ENSEMBLE.

To assess the influence of various modalities on clustering performance, this study undertakes a comprehensive exploration of feature selection strategies in ST. Through an ablation study, the contribution of gene expression profiles, spatial coordinates, neighborhood context, and image-derived morphological features is evaluated. This initial analysis enables the identification of the optimal combination of features necessary for downstream tasks. It highlights the importance of feature engineering in ST workflows. It also emphasizes the need for context-aware integration strategies. Our analysis clearly indicated that no single modality provides universally superior performances, underlining the synergistic effect of integrating diverse information for accurate spatial domain identification.

Following this, we proposed a set of optimized methods—Scatter, ACT, FACT, and an Ensemble strategy—developed to leverage these insights from the feature ablation study. The Scatter technique removes highly frequent genes from randomly selected spots. It then uses the remaining gene-count features to train an autoencoder. In contrast, the ACT technique first trains an autoencoder on the full gene-count matrix. It then refines the cluster assignments through joint optimization with a clustering layer that incorporates spatial coordinates and a novel refinement strategy. The final cluster assignments are obtained using Mclust. FACT further enhances this by incorporating the filtration strategy from Scatter within the ACT framework to improve feature selection. The Ensemble approach executes Scatter, ACT, FACT, GraphST, and STAGATE independently, selecting the result using a multi-metric selector as the final output, offering a robust, trainable and adaptive solution.

Empirical results across different configurations of the proposed framework reveal that the ensemble model consistently achieves the highest average ARI across samples and datasets. ACT also shows strong and consistent accuracy, particularly in recovering anatomically meaningful spatial domains. These results underscore the advantages of the proposed context-aware frameworks and demonstrate the effectiveness of joint spatial and gene-based clustering refinements.

On the DLPFC dataset, which consists of 12 spatially-resolved human brain tissue sections, the proposed method achieves the highest median clustering performance. It also demonstrates superior capability in distinctly separating adjacent cortical layers and reducing boundary mixing, which are commonly observed challenges in spatial domain analysis. This performance is further reinforced through clustering visualizations that align well with known anatomical structures, supporting the quantitative improvements observed in ARI metrics.

The superior performance of the Ensemble and ACT methods can be attributed to their integration of spatial information with gene expression, as well as the refinement strategies embedded in their architectures. The Ensemble method benefits from its ability to adaptively select the most suitable result based on silhouette width, while ACT’s success lies in its spatially informed optimization process that refines clustering outcomes beyond what gene expression alone can achieve. Overall, these methods demonstrate the necessity of both robust feature selection and spatially-aware clustering strategies to maximize accuracy in spatial transcriptomic analyses.

**Figure 12:**
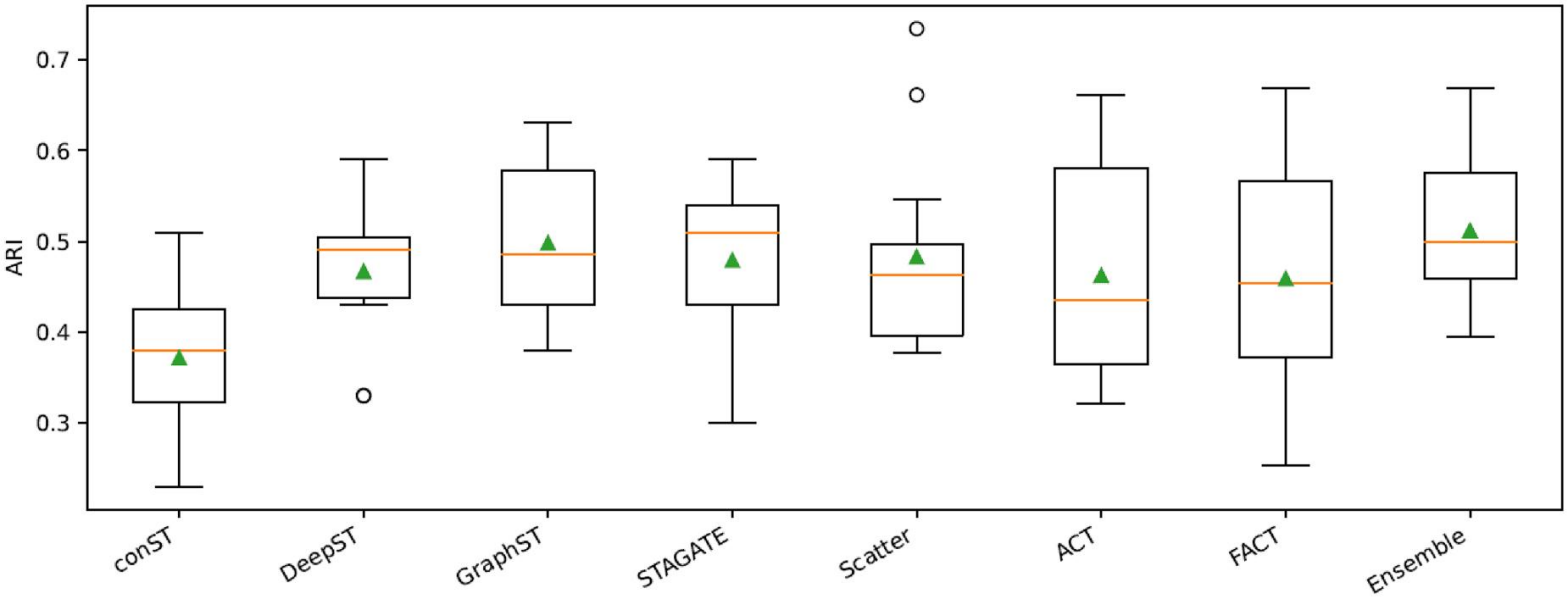
Distribution of ARI across the 12 DLPFC slides for representative baselines and proposed variants. The Ensemble shows the highest mean ARI and reduced variance relative to individual variants.

The single-metric ensemble variants also provide insight into the relative importance of the unsupervised criteria. On DLPFC, ASW-only selection achieves the highest mean ARI, suggesting that cluster separation/compactness is highly informative for this benchmark. However, the full five-metric Ensemble yields lower variance across slides, indicating that the additional spatial and structural metrics improve robustness by reducing brittle selections driven by a single criterion. Therefore, ASW appears to be the most discriminative individual metric on DLPFC, whereas PAS, CHAOS, Moran’s I, and Geary’s C mainly act as stabilizing constraints in the combined selector. Nevertheless, because no unsupervised metric is perfectly aligned with annotation-based ARI, occasional slide-level misselection remains possible. This trend is consistent with Fig. 12, where the ARI distribution across the 12 DLPFC slides is shown.

ST is a groundbreaking experimental approach that captures both gene expression profiles and their spatial arrangement within a tissue. By combining this dual modality, ST enables researchers to perform spatially informed clustering, revealing biologically coherent domains with substantial accuracy. ST sequencing enables a deeper understanding of complex biological systems by preserving spatial context alongside gene expression data. However, ST has technological constraints which can mitigate through computational methods.

Currently, approaches for identifying spatial domains fall into two categories: non-spatial and spatial clustering. Traditional non-spatial methods like *K*-means and Louvain cluster based solely on gene expression, often missing tissue structure. On the other hand, spatial clustering integrates gene expression, spatial location, and morphology to better capture spatial dependencies and align with tissue architecture.

As we have experimented, clustering solely based on morphological image data yielded inadequate performance despite the use of pre-trained ResNet50 embeddings. Moreover, direct concatenation of image features with gene-count data adversely affected the performance of clustering. Results suggest that image features should be integrated using alternative strategies rather than simple feature merging.

While the proposed framework demonstrates promising results, several aspects warrant further exploration. The computational cost of meta-heuristic optimization, though mitigated by our implementation, may still pose challenges for extremely large datasets. Also, applying our methods to imaging-based platforms some limitations may be faced. While the proposed framework is expected to be broadly applicable because it operates on spatially indexed expression profiles, its current empirical validation is limited to Visium, Slide-seqV2, and Stereo-seq. Imaging-based platforms such as MERFISH, seqFISH, and STARmap differ in several important respects: they often provide single-cell or subcellular spatial resolution, use targeted rather than transcriptome-wide panels, and depend on transcript decoding and cell segmentation for downstream expression quantification. These properties may affect the behavior of our framework in two ways. First, neighborhood refinement may need adaptive graph construction to avoid over-smoothing fine cellular boundaries. Second, filtration-based variants such as Scatter and FACT may require weaker or panel-aware filtering when the number of profiled genes is limited. We therefore expect the framework—especially the ACT variant—to remain applicable in principle, but its performance on imaging-based data should be regarded as a hypothesis supported by method design rather than a claim established by direct benchmarking. Another limitation is for the mouse brain anterior benchmark for which the original 52-region annotation was merged into 12 broader classes for detailed comparison with previous methods. While this improves interpretability and comparability, it also reduces sensitivity to fine anatomical boundaries, potentially favoring methods that produce smoother coarse domains and penalizing methods that recover finer biologically meaningful substructure.

In the future, dedicated evaluation on MERFISH, seqFISH, and STARmap datasets can be a way forward. We can also investigate parallelization strategies or alternative optimization algorithms to enhance scalability. Additionally, the generalization of the framework across diverse tissue types remains to be fully validated.

## Supplementary Material

vbag133_Supplementary_Data

## Data Availability

The data and source code is available on the GitHub repository: https://github.com/SababAosaf/FACTSpatialTranscriptomics.

## References

[vbag133-B1] 10x Genomics. Mouse Brain Serial Section 1 (Sagittal-Anterior). Spatial Gene Expression dataset analyzed using Space Ranger 1.1.0. Date published: 2020a. 06-23. https://www.10xgenomics.com/datasets/mouse-brain-serial-section-1-sagittal-anterior-1-standard-1-1-0 (27 December 2025, date last accessed).

[vbag133-B2] 10x Genomics. Human Breast Cancer (Block A, Section 1). Visium Spatial Gene Expression dataset, analyzed using Space Ranger 1.1.0, published 2020b https://www.10xgenomics.com/datasets/human-breast-cancer-block-a-section-1-1-standard-1-1-0 (23 May 2026, date last accessed).

[vbag133-B3] Batool F , Hennig C. Clustering with the average silhouette width. Comput Stat Data Anal 2021;158:107190. 10.1016/j.csda.2021.107190

[vbag133-B907258] Chen AO, , Liao S, , Cheng M et al Spatiotemporal transcriptomic atlas of mouse organogenesis using DNA nanoball-patterned arrays. Cell 2022;185:1777–92.e21. 10.1016/j.cell.2022.04.003 35512705

[vbag133-B5] Dong K , Zhang S. Deciphering spatial domains from spatially resolved transcriptomics with an adaptive graph attention auto-encoder. Nat Commun 2022;13:1739. 10.1038/s41467-022-29439-6 35365632 PMC8976049

[vbag133-B6] Geary RC. The contiguity ratio and statistical mapping. Incorporated Stat 1954;5:115–46.

[vbag133-B7] Halpern KB , Shenhav R, Matcovitch-Natan O et al Single-cell spatial reconstruction reveals global division of labour in the mammalian liver. Nature 2017;542:352–6. 10.1038/nature21065 28166538 PMC5321580

[vbag133-B8] He K , Zhang X, Ren S et al Deep residual learning for image recognition. In: *Proceedings of the IEEE Conference on Computer Vision and Pattern Recognition (CVPR).* Las Vegas, NV: IEEE, 2016:770–8. 10.1109/CVPR.2016.90

[vbag133-B9] Hu J , Li X, Coleman K et al SpaGCN: integrating gene expression, spatial location and histology to identify spatial domains and spatially variable genes by graph convolutional network. Nat Methods 2021;18:1342–51. 34711970 10.1038/s41592-021-01255-8

[vbag133-B10] Hu Y , Xie M, Li Y et al Benchmarking clustering, alignment, and integration methods for spatial transcriptomics. Genome Biol 2024;25:212. 10.1186/s13059-024-03361-0 39123269 PMC11312151

[vbag133-B11] Hubert L , Arabie P. Comparing partitions. J Classif 1985;2:193–218.

[vbag133-B12] Jain AK , Murty MN, Flynn PJ. Data clustering: a review. ACM Comput Surv 1999;31:264–323.

[vbag133-B13] Khosla P , Teterwak P, Wang C et al Supervised contrastive learning. Adv Neural Inf Process Syst 2020;33:18661–73.

[vbag133-B14] Kipf TN , Welling M. Variational graph auto-encoders. arXiv preprint, arXiv:1611.07308, 2016, preprint: not peer reviewed.

[vbag133-B15] Kvålseth TO. On normalized mutual information: measure derivations and properties. Entropy 2017;19:631.

[vbag133-B16] Liu T , Fang Z, Li X et al Assembling spatial clustering framework for heterogeneous spatial transcriptomics data with GRAPHDeep. Bioinformatics 2024;40:btae023. 38243703 10.1093/bioinformatics/btae023 PMC10832355

[vbag133-B17] Liu Y , Jin M, Pan S, et al Graph self-supervised learning: a survey. IEEE Trans Knowl Data Eng 2022;35:1–5900. 10.1109/TKDE.2022.3172903

[vbag133-B18] Long Y , Ang KS, Li M et al Spatially informed clustering, integration, and deconvolution of spatial transcriptomics with GraphST. Nat Commun 2023;14:1155. 36859400 10.1038/s41467-023-36796-3 PMC9977836

[vbag133-B19] Maynard KR , Collado-Torres L, Weber LM et al Transcriptome-scale spatial gene expression in the human dorsolateral prefrontal cortex. Nat Neurosci 2021;24:425–36. 33558695 10.1038/s41593-020-00787-0 PMC8095368

[vbag133-B20] Mclachlan GJ , Peel D. Finite Mixture Models 2000. New York: John Wiley & Sons.

[vbag133-B21] Meizlish ML , Franklin RA, Zhou X et al Tissue homeostasis and inflammation. Annu Rev Immunol 2021;39:557–81. 33651964 10.1146/annurev-immunol-061020-053734

[vbag133-B22] Moon TK. The expectation-maximization algorithm. IEEE Signal Process Mag 1996;13:47–60.

[vbag133-B23] Moran PAP. Notes on continuous stochastic phenomena. Biometrika 1950;37:17–23. 10.1093/biomet/37.1-2.17 15420245

[vbag133-B24] Pham D , Tan X, Balderson B et al Robust mapping of spatiotemporal trajectories and cell-cell interactions in healthy and diseased issues. Nat Commun 2023;14:7739. 10.1038/s41467-023-43120-6 38007580 PMC10676408

[vbag133-B25] Que X , Checconi F, Petrini F et al Scalable community detection with the Louvain algorithm. In: Proceedings of the 2015 IEEE International Parallel and Distributed Processing Symposium. Piscataway, NJ, USA: IEEE Computer Society;2015:28–37.

[vbag133-B26] Rahman MN , Noman AA, Turza AM et al ScribbleDom: using scribble-annotated histology images to identify domains in spatial transcriptomics data. Bioinformatics 2023;39:btad594. 37756699 10.1093/bioinformatics/btad594 PMC10564617

[vbag133-B27] Ren H , Walker BL, Cang Z et al Identifying multicellular spatiotemporal organization of cells with SpaceFlow. Nat Commun 2022;13:4076. 35835774 10.1038/s41467-022-31739-w PMC9283532

[vbag133-B28] Rodriques SG , Stickels RR, Goeva A et al Slide-seq: a scalable technology for measuring genome-wide expression at high spatial resolution. Science (1979) 2019;363:1463–7. 10.1126/science.aaw1219 PMC6927209 30923225

[vbag133-B29] Santos JM , Embrechts M. On the use of the adjusted rand index as a metric for evaluating supervised classification. In: International Conference on Artificial Neural Networks. Berlin, Heidelberg: Springer;2009:175–184.

[vbag133-B30] Scrucca L , Fraley C, Murphy TB, et al Model-based clustering, classification, and Density estimation. Using mclust in R. Boca Raton, FL: Chapman and Hall/CRC, 2023.

[vbag133-B31] Shlens J. Notes on Kullback–Leibler divergence and likelihood. arXiv preprint, arXiv:1404.2000, 2014, preprint: not peer reviewed.

[vbag133-B32] Silverman BW. Density Estimation for Statistics and Data Analysis. Milton Park, Abingdon, Oxon: Routledge, 2018.

[vbag133-B33] Stickels RR , Murray E, Kumar P-A et al Highly sensitive spatial transcriptomics at near-cellular resolution with Slide-seqV2. Nat Biotechnol 2021;39:313–9. 10.1038/s41587-020-0739-1 33288904 PMC8606189

[vbag133-B34] Stuart T , Butler A, Hoffman P et al Comprehensive integration of single-cell data. Cell 2019;177:1888–902.e21. 10.1016/j.cell.2019.05.031 31178118 PMC6687398

[vbag133-B35] Traag VA , Waltman L, Eck NJV. From Louvain to Leiden: guaranteeing well-connected communities. Sci Rep 2019;9:5233. 30914743 10.1038/s41598-019-41695-z PMC6435756

[vbag133-B36] van de Schoot R , Depaoli S, King R et al Bayesian statistics and modelling. Nat Rev Methods Primers 2021;1:1.

[vbag133-B37] Wu Z , Pan S, Chen F et al A comprehensive survey on graph neural networks. IEEE Trans Neural Netw Learn Syst 2021;32:4–24. 10.1109/TNNLS.2020.2978386 32217482

[vbag133-B38] Xu C , Jin X, Wei S et al DeepST: identifying spatial domains in spatial transcriptomics by deep learning. Nucleic Acids Res 2022;50:e131. 36250636 10.1093/nar/gkac901 PMC9825193

[vbag133-B39] Yin P , Yan X, Zhou J et al DGI: An easy and efficient framework for GNN model evaluation. In: *Proceedings of the 29th ACM SIGKDD Conference on Knowledge Discovery and Data Mining*. New York, NY: Association for Computing Machinery; 2023: 5439–5450.

[vbag133-B40] Zhang S , Tong H, Xu J et al Graph convolutional networks: a comprehensive review. Comput Soc Netw 2019;6:11–23. 37915858 10.1186/s40649-019-0069-y PMC10615927

[vbag133-B41] Zhao E , Stone MR, Ren X et al Spatial transcriptomics at subspot resolution with BayesSpace. Nat Biotechnol 2021;39:1375–84. 34083791 10.1038/s41587-021-00935-2 PMC8763026

[vbag133-B42] Zong Y , Yu T, Wang X et al conST: an interpretable multi-modal contrastive learning framework for spatial transcriptomics 2022:bioRxiv. 10.1101/2022.01.14.476408, preprint: not peer reviewed.

