# Supplementary material for: Trainable clustering framework for spatial transcriptomics
Source: Bioinform Adv. 2026 May 13;6(1):vbag133. doi: 10.1093/bioadv/vbag133 (PMC13242294; doi:10.1093/bioadv/vbag133)
Supplement: vbag133_Supplementary_Data [file vbag133_supplementary_data.zip › supplementary_document.pdf]

# Supplementary Document

# Trainable Clustering Framework for Spatial Transcriptomics

Riasat Azim[1,*], Sabab Aosaf[2], Swakkhar Shatabda[3],Mohammad Sohel Rahman[2], Salekul Islam[4],

[1]Department of Computer Science and Engineering, United International University,1212, Dhaka, Bangladesh
[2]Department of Computer Science and Engineering, Bangladesh University of Engineering and Technology,1000, Dhaka, Bangladesh
[3]Department of Computer Science and Engineering, Brac University,1212, Dhaka, Bangladesh
[4]Department of Electrical and Computer Engineering, North South University,1229, Dhaka, Bangladesh

*Corresponding author: riasat@cse.uiu.ac.bd

## Supplementary Equations

**S1.Homogeneity Score**
The homogeneity score measures how well each cluster contains only members of a single class. A high homogeneity score means that clusters contain only data points that are of a single class (Vysala and Gomes, 2020).

$$\text{Homogeneity} = 1 - \frac{H(C|K)}{H(C)} \tag{1}$$

$$H(C|K) = -\sum_{c\in C}\sum_{k\in K}\frac{|c\cap k|}{N}\log\left(\frac{|c\cap k|}{|k|}\right) \tag{2}$$

$$H(C) = -\sum_{c\in C}\frac{|c|}{N}\log\left(\frac{|c|}{N}\right) \tag{3}$$

- $H(C|K)$: Conditional entropy of the classes $C$ given the cluster assignments $K$.
- $H(C)$: Entropy of the classes $C$.
- $C$: The set of classes.
- $K$: The set of clusters.
- $N$: The total number of samples.
- $|c|$: The number of samples in class $c$.
- $|c\cap k|$: The number of samples in both class $c$ and cluster $k$.
- $|k|$: The number of samples in cluster $k$.
- log: The natural logarithm.

**S2.Completeness Score**
The completeness score is a metric used to evaluate how effectively the members of a given class are grouped into the same cluster. A high completeness score indicates that the members of each class are predominantly within a single cluster (Vysala and Gomes, 2020).

$$\text{Completeness} = 1 - \frac{H(K|C)}{H(K)} \tag{4}$$

$$H(K|C) = -\sum_{k\in K}\sum_{c\in C}\frac{|c\cap k|}{N}\log\left(\frac{|c\cap k|}{|c|}\right) \tag{5}$$

$$H(K) = -\sum_{k \in K} \frac{|k|}{N} \log\left(\frac{|k|}{N}\right) \tag{6}$$

- $H(K|C)$: Conditional entropy of the clusters $K$ given the class assignments $C$.
- $H(K)$: Entropy of the clusters $K$.
- $C$: The set of classes.
- $K$: The set of clusters.
- $N$: The total number of samples.
- $|c|$: The number of samples in class $c$.
- $|c \cap k|$: The number of samples in both class $c$ and cluster $k$.
- $|k|$: The number of samples in cluster $k$.
- log: The natural logarithm.

### S3.CHAOS score

The CHAOS score evaluates the spatial continuity of identified clusters by computing the mean length of the graph edges in a 1-nearest neighbor (1NN) graph for each cluster. A lower CHAOS score signifies improved clustering performance (Hu et al., 2024).

$$\text{Homogeneity} = 1 - \frac{H(C|K)}{H(C)} \tag{7}$$

$$\text{Accuracy} = \frac{\text{True Positives} + \text{True Negatives}}{\text{Total Samples}} \tag{8}$$

$$\text{CHAOS} = \alpha \times \text{Homogeneity} + \beta \times \text{Accuracy} \tag{9}$$

- $H(C|K)$: Conditional entropy of the classes $C$ given the cluster assignments $K$.
- $H(C)$: Entropy of the classes $C$.
- $\alpha$: Weight for homogeneity.
- $\beta$: Weight for accuracy.
- True Positives: Number of true positive samples.
- True Negatives: Number of true negative samples.
- Total Samples: Total number of samples.

$$H(C|K) = -\sum_{c \in C} \sum_{k \in K} \frac{|c \cap k|}{N} \log\left(\frac{|c \cap k|}{|k|}\right) \tag{10}$$

$$H(C) = -\sum_{c \in C} \frac{|c|}{N} \log\left(\frac{|c|}{N}\right) \tag{11}$$

- $C$: The set of classes.
- $K$: The set of clusters.
- $N$: The total number of samples.
- $|c|$: The number of samples in class $c$.
- $|c \cap k|$: The number of samples in both class $c$ and cluster $k$.
- $|k|$: The number of samples in cluster $k$.
- log: The natural logarithm.

### S4.PAS score

The PAS score is used to evaluate clustering in spatial domains. It measures the randomness of spots located outside the spatial region of their cluster. A lower PAS score may mean better clustering (Hu et al., 2024).

$$\text{PAS} = \frac{\sum_{i=1}^{N} \sum_{j=1}^{N} \mathbb{1}(x_i \in C_j) \cdot \mathbb{1}(x_j \in C_i)}{N(N-1)} \tag{12}$$

- $N$: Total number of samples.
- $x_i$: The $i$-th sample.
- $C_j$: The $j$-th cluster.
- $\mathbb{1}(x_i \in C_j)$: Indicator function that is 1 if sample $x_i$ is in cluster $C_j$, and 0 otherwise.

**S5.The Average Silhouette Width (ASW)**

The Average Silhouette Width (ASW) is used to find the optimal number of clusters in a dataset. It evaluates the degree to which each data point is grouped within its cluster. Higher ASW scores indicates well-defined clusters (Batool and Hennig, 2021).

$$\text{ASW} = \frac{1}{N} \sum_{i=1}^{N} s(i) \tag{13}$$

- $s(i)$: Silhouette width of the $i$-th sample.
- $N$: Total number of samples.

The silhouette width for a single sample i is calculated as:

$$s(i) = \frac{b(i) - a(i)}{\max(a(i), b(i))} \tag{14}$$

- $a(i)$: The average distance from the $i$-th sample to all other points in the same cluster.
- $b(i)$: The minimum average distance from the $i$-th sample to all points in any other cluster, of which $i$ is not a member.

**S6.The Moran's I score** The Moran's I score assesses whether the expression of a gene is clustered, dispersed, or random across. A higher Moran's I score indicates clustering, while a lower score indicates dispersion (Moran, 1950).

$$I = \frac{N \sum_{i=1}^{N} \sum_{j=1}^{N} w_{ij}(x_i - \bar{x})(x_j - \bar{x})}{S_0 \sum_{i=1}^{N} (x_i - \bar{x})^2} \tag{15}$$

- $I$: Moran's I statistic.
- $N$: Total number of observations.
- $x_i$: Value of the variable at location $i$.
- $\bar{x}$: Mean of the variable.
- $w_{ij}$: Spatial weight between location $i$ and $j$.
- $S_0$: Sum of all spatial weights, $S_0 = \sum_{i=1}^{N} \sum_{j=1}^{N} w_{ij}$.

**S7.Geary's C Score**

Geary's C Score evaluates the degree to which a variable is correlated with itself over space and gives idea about clustering or dispersion of spatial (Geary, 1954).

$$C = \frac{(N-1) \sum_{i=1}^{N} \sum_{j=1}^{N} w_{ij}(x_i - x_j)^2}{2S_0 \sum_{i=1}^{N} (x_i - \bar{x})^2} \tag{16}$$

- $C$: Geary's C statistic.
- $N$: Total number of observations.
- $x_i$: Value of the variable at location $i$.

- $x_j$: Value of the variable at location $j$.
- $\bar{x}$: Mean of the variable.
- $w_{ij}$: Spatial weight between location $i$ and $j$.
- $S_0$: Sum of all spatial weights, $S_0 = \sum_{i=1}^{N} \sum_{j=1}^{N} w_{ij}$.

## Supplementary Tables

**Table 1.** Normalized Mutual Information (NMI) values of clustering methods on the DLPFC dataset

| Method | 507 | 508 | 509 | 510 | 669 | 670 | 671 | 672 | 673 | 674 | 675 | 676 |
|---|---|---|---|---|---|---|---|---|---|---|---|---|
| **conST** | 0.42 | 0.50 | 0.61 | 0.50 | 0.57 | 0.46 | 0.57 | 0.59 | 0.66 | 0.65 | 0.58 | 0.55 |
| **DeepST** | 0.65 | 0.59 | 0.64 | 0.61 | 0.55 | 0.53 | 0.65 | 0.64 | 0.71 | 0.60 | 0.67 | 0.67 |
| **SpaceFlow** | 0.47 | 0.41 | 0.40 | 0.38 | 0.43 | 0.38 | 0.42 | 0.44 | 0.50 | 0.44 | 0.38 | 0.41 |
| **STAGATE** | 0.69 | 0.58 | 0.59 | 0.59 | 0.55 | 0.64 | 0.46 | 0.66 | 0.63 | 0.67 | 0.67 | 0.63 |
| **SpaGCN** | 0.54 | 0.50 | 0.54 | 0.56 | 0.45 | 0.44 | 0.56 | 0.61 | 0.63 | 0.53 | 0.50 | 0.50 |
| **GraphST** | 0.65 | 0.64 | 0.62 | 0.65 | 0.60 | 0.55 | 0.72 | 0.70 | 0.73 | 0.61 | 0.62 | 0.66 |
| **G** | 0.67 | 0.00 | 0.62 | 0.61 | 0.58 | 0.57 | 0.69 | 0.68 | 0.73 | 0.69 | 0.62 | 0.69 |
| **GS** | 0.66 | 0.55 | 0.62 | 0.65 | 0.61 | 0.57 | 0.71 | 0.68 | 0.69 | 0.63 | 0.67 | 0.69 |
| **I** | 0.16 | 0.23 | 0.23 | 0.21 | 0.13 | 0.13 | 0.20 | 0.18 | 0.25 | 0.30 | 0.22 | 0.27 |
| **IG** | 0.19 | 0.24 | 0.22 | 0.21 | 0.13 | 0.16 | 0.21 | 0.28 | 0.34 | 0.34 | 0.23 | 0.32 |
| **IS** | 0.19 | 0.23 | 0.23 | 0.21 | 0.13 | 0.14 | 0.18 | 0.15 | 0.31 | 0.27 | 0.25 | 0.25 |
| **IGS** | 0.24 | 0.24 | 0.25 | 0.21 | 0.14 | 0.18 | 0.23 | 0.29 | 0.36 | 0.32 | 0.26 | 0.30 |
| **GSD** | 0.63 | 0.57 | 0.62 | 0.63 | 0.66 | 0.57 | 0.68 | 0.67 | 0.71 | 0.61 | 0.62 | 0.59 |
| **GD** | 0.65 | 0.65 | 0.61 | 0.60 | 0.53 | 0.57 | 0.67 | 0.68 | 0.62 | 0.59 | 0.62 | 0.69 |
| **GN** | 0.65 | 0.57 | 0.64 | 0.61 | 0.53 | 0.56 | 0.71 | 0.68 | 0.73 | 0.68 | 0.65 | 0.64 |
| **Scatter** | 0.63 | 0.54 | 0.64 | 0.55 | 0.60 | 0.56 | 0.67 | 0.71 | 0.74 | 0.68 | 0.60 | 0.57 |
| **ACT** | 0.62 | 0.52 | 0.61 | 0.43 | 0.58 | 0.54 | 0.73 | 0.68 | 0.73 | 0.68 | 0.50 | 0.58 |
| **FACT** | 0.67 | 0.47 | 0.65 | 0.57 | 0.60 | 0.55 | 0.72 | 0.69 | 0.73 | 0.62 | 0.43 | 0.46 |
| **Ensemble** | 0.60 | 0.48 | 0.61 | 0.61 | 0.61 | 0.54 | 0.69 | 0.61 | 0.74 | 0.69 | 0.62 | 0.64 |

**Table 2.** HOM values of clustering methods on the DLPFC dataset

| Method | 507 | 508 | 509 | 510 | 669 | 670 | 671 | 672 | 673 | 674 | 675 | 676 |
|---|---|---|---|---|---|---|---|---|---|---|---|---|
| **conST** | 0.42 | 0.51 | 0.66 | 0.54 | 0.68 | 0.60 | 0.67 | 0.69 | 0.67 | 0.64 | 0.59 | 0.56 |
| **DeepST** | 0.64 | 0.57 | 0.61 | 0.57 | 0.46 | 0.44 | 0.58 | 0.58 | 0.69 | 0.62 | 0.67 | 0.66 |
| **SpaceFlow** | 0.46 | 0.40 | 0.37 | 0.35 | 0.36 | 0.30 | 0.36 | 0.40 | 0.48 | 0.43 | 0.38 | 0.41 |
| **STAGATE** | 0.69 | 0.58 | 0.59 | 0.58 | 0.54 | 0.64 | 0.46 | 0.65 | 0.63 | 0.67 | 0.67 | 0.62 |
| **SpaGCN** | 0.56 | 0.49 | 0.51 | 0.53 | 0.40 | 0.35 | 0.49 | 0.56 | 0.61 | 0.52 | 0.50 | 0.50 |
| **GraphST** | 0.68 | 0.64 | 0.63 | 0.66 | 0.52 | 0.46 | 0.65 | 0.64 | 0.72 | 0.61 | 0.62 | 0.65 |
| **G** | 0.67 | 0 | 0.68 | 0.8 | 0.49 | 0.48 | 0.62 | 0.65 | 0.75 | 0.7 | 0.67 | 0.72 |
| **GS** | 0.67 | 0.60 | 0.68 | 0.71 | 0.53 | 0.48 | 0.65 | 0.64 | 0.73 | 0.70 | 0.71 | 0.72 |
| **I** | 0.17 | 0.27 | 0.25 | 0.22 | 0.15 | 0.12 | 0.20 | 0.24 | 0.42 | 0.39 | 0.27 | 0.40 |
| **IG** | 0.24 | 0.28 | 0.29 | 0.23 | 0.12 | 0.18 | 0.20 | 0.34 | 0.42 | 0.39 | 0.28 | 0.36 |
| **IS** | 0.24 | 0.27 | 0.25 | 0.23 | 0.14 | 0.14 | 0.17 | 0.18 | 0.46 | 0.33 | 0.29 | 0.38 |
| **IGS** | 0.26 | 0.28 | 0.27 | 0.22 | 0.16 | 0.21 | 0.22 | 0.29 | 0.44 | 0.38 | 0.28 | 0.34 |
| **GSD** | 0.67 | 0.62 | 0.69 | 0.63 | 0.60 | 0.48 | 0.61 | 0.64 | 0.70 | 0.66 | 0.66 | 0.62 |
| **GD** | 0.67 | 0.66 | 0.62 | 0.62 | 0.48 | 0.48 | 0.60 | 0.65 | 0.71 | 0.60 | 0.67 | 0.72 |
| **GN** | 0.71 | 0.62 | 0.65 | 0.61 | 0.48 | 0.47 | 0.65 | 0.65 | 0.75 | 0.69 | 0.71 | 0.66 |
| **Scatter** | 0.66 | 0.59 | 0.63 | 0.61 | 0.52 | 0.47 | 0.58 | 0.70 | 0.75 | 0.70 | 0.66 | 0.59 |
| **ACT** | 0.65 | 0.65 | 0.63 | 0.49 | 0.52 | 0.47 | 0.68 | 0.65 | 0.75 | 0.70 | 0.56 | 0.67 |
| **FACT** | 0.74 | 0.59 | 0.72 | 0.66 | 0.52 | 0.48 | 0.68 | 0.66 | 0.75 | 0.68 | 0.50 | 0.57 |
| **Ensemble** | 0.67 | 0.52 | 0.63 | 0.61 | 0.54 | 0.46 | 0.62 | 0.59 | 0.75 | 0.63 | 0.67 | 0.66 |

**Table 3.** CM values of clustering methods on the DLPFC dataset

| Method | 507 | 508 | 509 | 510 | 669 | 670 | 671 | 672 | 673 | 674 | 675 | 676 |
|---|---|---|---|---|---|---|---|---|---|---|---|---|
| **conST** | 0.42 | 0.49 | 0.57 | 0.47 | 0.48 | 0.37 | 0.50 | 0.52 | 0.65 | 0.65 | 0.58 | 0.54 |
| **DeepST** | 0.65 | 0.61 | 0.67 | 0.65 | 0.69 | 0.67 | 0.75 | 0.71 | 0.73 | 0.58 | 0.67 | 0.68 |
| **SpaceFlow** | 0.48 | 0.43 | 0.44 | 0.42 | 0.55 | 0.50 | 0.50 | 0.50 | 0.52 | 0.45 | 0.39 | 0.42 |
| **STAGATE** | 0.69 | 0.58 | 0.59 | 0.59 | 0.56 | 0.64 | 0.46 | 0.67 | 0.63 | 0.67 | 0.68 | 0.63 |
| **SpaGCN** | 0.52 | 0.52 | 0.57 | 0.60 | 0.52 | 0.59 | 0.67 | 0.67 | 0.65 | 0.54 | 0.51 | 0.51 |
| **GraphST** | 0.63 | 0.64 | 0.62 | 0.65 | 0.70 | 0.68 | 0.80 | 0.78 | 0.75 | 0.61 | 0.61 | 0.66 |
| **G** | 0.66 | 0.00 | 0.56 | 0.50 | 0.70 | 0.70 | 0.77 | 0.71 | 0.71 | 0.68 | 0.57 | 0.66 |
| **GS** | 0.65 | 0.52 | 0.57 | 0.59 | 0.74 | 0.70 | 0.78 | 0.71 | 0.66 | 0.58 | 0.63 | 0.66 |
| **I** | 0.14 | 0.20 | 0.21 | 0.19 | 0.12 | 0.14 | 0.20 | 0.15 | 0.18 | 0.25 | 0.19 | 0.20 |
| **IG** | 0.16 | 0.20 | 0.18 | 0.20 | 0.13 | 0.14 | 0.22 | 0.23 | 0.29 | 0.30 | 0.20 | 0.29 |
| **IS** | 0.16 | 0.20 | 0.21 | 0.19 | 0.12 | 0.15 | 0.18 | 0.13 | 0.24 | 0.22 | 0.22 | 0.19 |
| **IGS** | 0.23 | 0.21 | 0.23 | 0.20 | 0.12 | 0.15 | 0.24 | 0.28 | 0.31 | 0.27 | 0.24 | 0.27 |
| **GSD** | 0.60 | 0.53 | 0.57 | 0.63 | 0.74 | 0.70 | 0.77 | 0.71 | 0.72 | 0.57 | 0.58 | 0.56 |
| **GD** | 0.62 | 0.64 | 0.60 | 0.59 | 0.59 | 0.70 | 0.75 | 0.72 | 0.54 | 0.58 | 0.57 | 0.66 |
| **GN** | 0.60 | 0.52 | 0.62 | 0.61 | 0.59 | 0.70 | 0.78 | 0.71 | 0.71 | 0.67 | 0.60 | 0.61 |
| **Scatter** | 0.60 | 0.50 | 0.64 | 0.50 | 0.70 | 0.70 | 0.78 | 0.72 | 0.72 | 0.66 | 0.55 | 0.55 |
| **ACT** | 0.60 | 0.43 | 0.59 | 0.39 | 0.65 | 0.64 | 0.78 | 0.72 | 0.71 | 0.67 | 0.45 | 0.51 |
| **FACT** | 0.61 | 0.40 | 0.60 | 0.50 | 0.70 | 0.66 | 0.77 | 0.71 | 0.71 | 0.58 | 0.38 | 0.39 |
| **Ensemble** | 0.54 | 0.45 | 0.58 | 0.61 | 0.69 | 0.64 | 0.77 | 0.63 | 0.72 | 0.68 | 0.57 | 0.61 |

**Table 4.** CHAOS values of clustering methods on the DLPFC dataset

| Method | 507 | 508 | 509 | 510 | 669 | 670 | 671 | 672 | 673 | 674 | 675 | 676 |
|---|---|---|---|---|---|---|---|---|---|---|---|---|
| **conST** | 0.06 | 0.06 | 0.05 | 0.06 | 0.06 | 0.07 | 0.06 | 0.06 | 0.06 | 0.06 | 0.06 | 0.07 |
| **DeepST** | 0.06 | 0.06 | 0.05 | 0.06 | 0.06 | 0.06 | 0.06 | 0.06 | 0.06 | 0.06 | 0.06 | 0.06 |
| **SpaceFlow** | 0.06 | 0.06 | 0.05 | 0.06 | 0.06 | 0.06 | 0.06 | 0.06 | 0.06 | 0.06 | 0.06 | 0.06 |
| **STAGATE** | 0.06 | 0.06 | 0.05 | 0.06 | 0.06 | 0.06 | 0.06 | 0.06 | 0.06 | 0.06 | 0.06 | 0.06 |
| **SpaGCN** | 0.06 | 0.06 | 0.06 | 0.06 | 0.07 | 0.06 | 0.06 | 0.06 | 0.06 | 0.06 | 0.06 | 0.06 |
| **GraphST** | 0.06 | 0.06 | 0.05 | 0.06 | 0.06 | 0.06 | 0.06 | 0.06 | 0.06 | 0.06 | 0.06 | 0.06 |
| **G** | 0.06 | 0.00 | 0.05 | 0.06 | 0.06 | 0.06 | 0.06 | 0.06 | 0.06 | 0.06 | 0.06 | 0.06 |
| **GS** | 0.06 | 0.06 | 0.05 | 0.06 | 0.06 | 0.06 | 0.06 | 0.06 | 0.06 | 0.06 | 0.06 | 0.06 |
| **I** | 0.06 | 0.06 | 0.05 | 0.06 | 0.06 | 0.06 | 0.06 | 0.06 | 0.06 | 0.06 | 0.06 | 0.06 |
| **IG** | 0.06 | 0.06 | 0.05 | 0.06 | 0.06 | 0.06 | 0.06 | 0.06 | 0.06 | 0.06 | 0.06 | 0.06 |
| **IS** | 0.06 | 0.06 | 0.05 | 0.06 | 0.06 | 0.06 | 0.06 | 0.06 | 0.06 | 0.06 | 0.06 | 0.06 |
| **IGS** | 0.06 | 0.06 | 0.05 | 0.06 | 0.06 | 0.06 | 0.06 | 0.06 | 0.06 | 0.06 | 0.06 | 0.06 |
| **GSD** | 0.06 | 0.06 | 0.05 | 0.06 | 0.06 | 0.06 | 0.06 | 0.06 | 0.06 | 0.06 | 0.06 | 0.06 |
| **GD** | 0.06 | 0.06 | 0.05 | 0.06 | 0.06 | 0.06 | 0.06 | 0.06 | 0.06 | 0.06 | 0.06 | 0.06 |
| **GN** | 0.06 | 0.06 | 0.05 | 0.06 | 0.06 | 0.06 | 0.06 | 0.06 | 0.06 | 0.06 | 0.06 | 0.06 |
| **Scatter** | 0.06 | 0.06 | 0.05 | 0.06 | 0.06 | 0.06 | 0.06 | 0.06 | 0.06 | 0.06 | 0.06 | 0.06 |
| **ACT** | 0.06 | 0.06 | 0.05 | 0.06 | 0.06 | 0.06 | 0.06 | 0.06 | 0.06 | 0.06 | 0.06 | 0.06 |
| **FACT** | 0.06 | 0.06 | 0.05 | 0.06 | 0.06 | 0.06 | 0.06 | 0.06 | 0.06 | 0.06 | 0.06 | 0.06 |
| **Ensemble** | 0.06 | 0.06 | 0.05 | 0.06 | 0.06 | 0.06 | 0.06 | 0.06 | 0.06 | 0.06 | 0.06 | 0.06 |

**Table 5.** PAS values of clustering methods on the DLPFC dataset

| Method | 507 | 508 | 509 | 510 | 669 | 670 | 671 | 672 | 673 | 674 | 675 | 676 |
|---|---|---|---|---|---|---|---|---|---|---|---|---|
| **conST** | 0.31 | 0.24 | 0.04 | 0.19 | 0.07 | 0.20 | 0.09 | 0.06 | 0.04 | 0.03 | 0.14 | 0.22 |
| **DeepST** | 0.07 | 0.05 | 0.05 | 0.05 | 0.04 | 0.09 | 0.05 | 0.05 | 0.03 | 0.03 | 0.04 | 0.07 |
| **SpaceFlow** | 0.01 | 0.02 | 0.02 | 0.02 | 0.03 | 0.03 | 0.01 | 0.03 | 0.03 | 0.04 | 0.02 | 0.02 |
| **STAGATE** | 0.04 | 0.03 | 0.04 | 0.04 | 0.04 | 0.04 | 0.03 | 0.05 | 0.03 | 0.03 | 0.03 | 0.04 |
| **SpaGCN** | 0.12 | 0.13 | 0.15 | 0.17 | 0.14 | 0.16 | 0.14 | 0.12 | 0.08 | 0.10 | 0.15 | 0.11 |
| **GraphST** | 0.01 | 0.01 | 0.01 | 0.01 | 0.01 | 0.02 | 0.02 | 0.02 | 0.02 | 0.02 | 0.02 | 0.02 |
| **G** | 0.03 | 0.00 | 0.02 | 0.01 | 0.02 | 0.01 | 0.02 | 0.01 | 0.01 | 0.01 | 0.01 | 0.02 |
| **GS** | 0.03 | 0.03 | 0.01 | 0.02 | 0.02 | 0.02 | 0.02 | 0.01 | 0.02 | 0.01 | 0.02 | 0.02 |
| **I** | 0.07 | 0.02 | 0.02 | 0.03 | 0.00 | 0.06 | 0.01 | 0.01 | 0.04 | 0.06 | 0.03 | 0.01 |
| **IG** | 0.02 | 0.03 | 0.01 | 0.03 | 0.05 | 0.01 | 0.04 | 0.01 | 0.02 | 0.04 | 0.03 | 0.05 |
| **IS** | 0.02 | 0.02 | 0.02 | 0.02 | 0.02 | 0.04 | 0.04 | 0.03 | 0.02 | 0.05 | 0.03 | 0.02 |
| **IGS** | 0.04 | 0.03 | 0.02 | 0.03 | 0.01 | 0.03 | 0.06 | 0.04 | 0.03 | 0.04 | 0.05 | 0.05 |
| **GSD** | 0.02 | 0.02 | 0.02 | 0.02 | 0.01 | 0.02 | 0.02 | 0.01 | 0.01 | 0.02 | 0.02 | 0.03 |
| **GD** | 0.02 | 0.01 | 0.02 | 0.02 | 0.01 | 0.02 | 0.02 | 0.01 | 0.01 | 0.02 | 0.01 | 0.02 |
| **GN** | 0.01 | 0.02 | 0.02 | 0.03 | 0.01 | 0.02 | 0.02 | 0.01 | 0.01 | 0.02 | 0.02 | 0.02 |
| **Scatter** | 0.02 | 0.03 | 0.02 | 0.02 | 0.01 | 0.02 | 0.02 | 0.02 | 0.01 | 0.02 | 0.02 | 0.02 |
| **ACT** | 0.02 | 0.01 | 0.02 | 0.04 | 0.01 | 0.02 | 0.01 | 0.01 | 0.01 | 0.02 | 0.04 | 0.01 |
| **FACT** | 0.01 | 0.02 | 0.01 | 0.02 | 0.02 | 0.01 | 0.01 | 0.01 | 0.01 | 0.01 | 0.04 | 0.02 |
| **Ensemble** | 0.02 | 0.05 | 0.03 | 0.03 | 0.01 | 0.02 | 0.02 | 0.02 | 0.01 | 0.01 | 0.01 | 0.02 |

**Table 6.** Total Execution time of different clustering methods on the DLPFC dataset-1

| Method | 507 | 508 | 509 | 510 | 669 | 670 | 671 |
|---|---|---|---|---|---|---|---|
| **conST** | 248.04 | 217.02 | 249.22 | 244.99 | 182.68 | 169.04 | 199.56 |
| **DeepST** | 701.62 | 592.93 | 725.46 | 774.12 | 547.64 | 515.74 | 550.65 |
| **SpaceFlow** | 79.09 | 50.75 | 38.31 | 50.23 | 28.95 | 26.95 | 34.16 |
| **STAGATE** | 75.20 | 41.09 | 51.24 | 48.44 | 49.58 | 58.02 | 114.90 |
| **SpaGCN** | 223.04 | 253.36 | 309.46 | 286.12 | 173.19 | 175.63 | 219.85 |
| **GraphST** | 73.11 | 65.80 | 92.56 | 87.64 | 64.64 | 64.95 | 54.50 |
| **G** | 86.20 | 0.00 | 62.33 | 64.51 | 60.61 | 49.28 | 54.15 |
| **GS** | 52.95 | 49.99 | 58.57 | 60.39 | 47.53 | 45.89 | 52.31 |
| **I** | 372.55 | 339.67 | 346.16 | 336.89 | 271.33 | 256.74 | 326.87 |
| **IG** | 331.60 | 395.00 | 412.25 | 393.62 | 317.05 | 309.97 | 317.20 |
| **IS** | 400.76 | 346.33 | 352.37 | 344.48 | 272.84 | 250.07 | 291.49 |
| **IGS** | 54.96 | 76.16 | 68.06 | 61.75 | 47.00 | 46.03 | 54.21 |
| **GSD** | 276.10 | 285.61 | 319.89 | 297.86 | 295.29 | 289.68 | 363.65 |
| **GD** | 275.80 | 278.79 | 302.65 | 306.14 | 227.15 | 220.49 | 274.19 |
| **GN** | 83.32 | 104.17 | 97.62 | 114.30 | 86.74 | 77.22 | 82.25 |
| **Scatter** | 180.27 | 198.39 | 199.36 | 185.77 | 175.88 | 200.11 | 186.12 |
| **ACT** | 326.26 | 339.46 | 358.00 | 361.07 | 293.35 | 281.94 | 300.66 |
| **FACT** | 459.33 | 454.97 | 480.61 | 503.85 | 412.88 | 376.56 | 406.95 |
| **Ensemble** | 669.52 | 619.25 | 707.09 | 733.12 | 630.65 | 562.07 | 617.33 |

**Table 7.** Total Execution time of different clustering methods on the DLPFC dataset-2

| Method | 672 | 673 | 674 | 675 | 676 |
|---|---|---|---|---|---|
| conST | 173.32 | 179.13 | 176.74 | 179.94 | 172.54 |
| DeepST | 528.57 | 519.91 | 528.27 | 514.97 | 454.36 |
| SpaceFlow | 30.81 | 21.76 | 39.71 | 41.90 | 36.57 |
| STAGATE | 44.14 | 37.84 | 39.37 | 38.10 | 38.23 |
| SpaGCN | 200.99 | 213.47 | 177.30 | 177.56 | 145.52 |
| GraphST | 49.45 | 53.50 | 65.86 | 64.11 | 58.00 |
| G | 56.16 | 45.09 | 47.34 | 50.45 | 50.85 |
| GS | 46.55 | 46.44 | 42.84 | 41.50 | 42.45 |
| I | 322.38 | 277.07 | 290.00 | 273.48 | 260.49 |
| IG | 315.76 | 321.23 | 318.65 | 312.39 | 303.79 |
| IS | 286.79 | 259.44 | 271.00 | 251.59 | 240.54 |
| IGS | 53.03 | 47.51 | 50.74 | 48.76 | 46.95 |
| GSD | 275.89 | 211.81 | 218.39 | 209.99 | 204.89 |
| GD | 251.47 | 224.20 | 219.87 | 225.26 | 229.24 |
| GN | 95.57 | 78.83 | 84.03 | 73.92 | 72.26 |
| Scatter | 185.08 | 176.21 | 173.18 | 173.84 | 164.62 |
| ACT | 305.35 | 301.94 | 299.26 | 289.15 | 295.22 |
| FACT | 417.35 | 385.24 | 409.12 | 393.38 | 374.51 |
| Ensemble | 613.85 | 576.81 | 590.36 | 567.28 | 571.62 |

**Table 8.** Space Taken by different clustering methods on the DLPFC dataset-1

| Method | 507 | 508 | 509 | 510 | 669 | 670 | 671 |
|---|---|---|---|---|---|---|---|
| conST | 753.24 | 825.56 | 927.87 | 888.84 | 768.43 | 727.20 | 860.63 |
| DeepST | 5835.28 | 6041.91 | 6603.86 | 6389.21 | 5077.73 | 4853.62 | 5700.06 |
| SpaceFlow | 334.05 | 328.38 | 385.77 | 420.04 | 283.26 | 263.59 | 321.39 |
| STAGATE | 337.34 | 379.19 | 379.06 | 379.06 | 379.06 | 379.06 | 379.06 |
| SpaGCN | 1453.12 | 1409.16 | 1558.82 | 1503.75 | 1311.93 | 1256.13 | 1500.47 |
| GraphST | 2599.23 | 2694.63 | 2945.88 | 2835.64 | 2209.03 | 2145.44 | 2510.53 |
| G | 3568.09 | 0.00 | 6675.46 | 3939.13 | 3091.82 | 2936.41 | 3504.03 |
| GS | 5453.35 | 5653.32 | 6283.64 | 6060.76 | 4767.98 | 4537.93 | 5385.76 |
| I | 2752.13 | 2832.10 | 3098.23 | 2997.03 | 2440.99 | 2340.27 | 2709.92 |
| IG | 4235.25 | 4370.67 | 4778.94 | 4623.33 | 3725.82 | 3567.91 | 4152.33 |
| IS | 2816.82 | 2899.20 | 3171.53 | 3067.95 | 2497.02 | 2393.81 | 2772.82 |
| IGS | 6105.28 | 6310.62 | 6952.69 | 6724.47 | 5345.84 | 5115.79 | 5971.04 |
| GSD | 5976.31 | 6176.86 | 6752.02 | 6532.57 | 5234.18 | 5009.11 | 5845.68 |
| GD | 11086.69 | 11488.84 | 12584.34 | 12165.14 | 9629.77 | 9200.30 | 10808.50 |
| GN | 7685.75 | 7960.74 | 8784.83 | 8435.79 | 6683.45 | 6385.15 | 7500.84 |
| Scatter | 11569.27 | 11989.60 | 13131.43 | 12693.91 | 10047.98 | 9600.25 | 11277.72 |
| ACT | 4005.87 | 4151.55 | 4586.33 | 4420.63 | 3459.69 | 3291.15 | 3915.48 |
| FACT | 4005.60 | 4151.22 | 4586.00 | 4420.13 | 3459.42 | 3291.00 | 3915.09 |
| Ensemble | 7685.75 | 7960.74 | 8784.83 | 8435.79 | 6683.45 | 6385.15 | 7500.84 |

**Table 9.** Space Taken by different clustering methods on the DLPFC dataset-2

| Method | 672 | 673 | 674 | 675 | 676 |
|---|---|---|---|---|---|
| **conST** | 827.50 | 805.62 | 867.08 | 760.38 | 748.32 |
| **DeepST** | 5557.55 | 5059.19 | 5119.19 | 4984.39 | 4809.13 |
| **SpaceFlow** | 307.81 | 390.17 | 454.92 | 280.63 | 286.16 |
| **STAGATE** | 379.06 | 379.06 | 379.06 | 379.06 | 379.06 |
| **SpaGCN** | 1397.39 | 1294.32 | 1430.05 | 1297.17 | 1277.36 |
| **GraphST** | 2440.51 | 2282.55 | 2346.91 | 2164.46 | 2085.87 |
| **G** | 3407.88 | 3105.77 | 3177.92 | 3030.72 | 2923.52 |
| **GS** | 5246.10 | 4771.86 | 4859.56 | 4675.29 | 4507.64 |
| **I** | 2646.80 | 2450.76 | 2498.18 | 2401.43 | 2332.40 |
| **IG** | 4055.87 | 3727.87 | 3787.24 | 3662.05 | 3546.70 |
| **IS** | 2708.25 | 2506.45 | 2554.40 | 2456.41 | 2385.36 |
| **IGS** | 5888.58 | 5401.44 | 5490.30 | 5251.52 | 5077.77 |
| **GSD** | 5710.08 | 5227.08 | 5300.54 | 5141.99 | 4972.26 |
| **GD** | 10552.38 | 9595.02 | 9711.22 | 9450.96 | 9115.90 |
| **GN** | 7321.18 | 6666.41 | 6755.34 | 6560.16 | 6331.36 |
| **Scatter** | 11011.42 | 10011.01 | 10131.06 | 9861.43 | 9511.87 |
| **ACT** | 3812.82 | 3460.21 | 3521.89 | 3391.42 | 3267.47 |
| **FACT** | 3812.65 | 3460.02 | 3521.69 | 3391.17 | 3267.41 |
| **Ensemble** | 7321.18 | 6666.41 | 6755.34 | 6560.16 | 6331.36 |

## Supplementary Figures

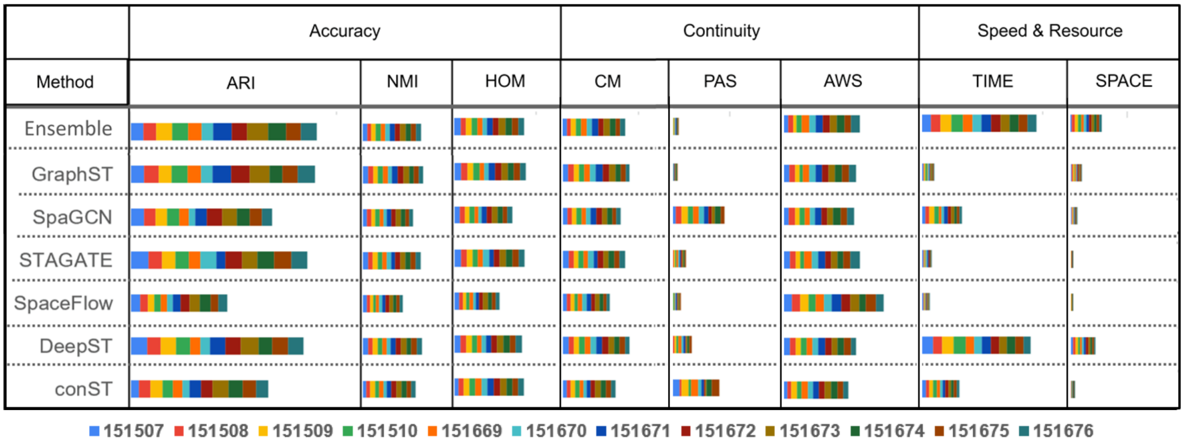


**Fig. 1.** Comparison of the proposed framework with state-of-the-art methods (GraphST, SpaGCN, STAGATE, SpaceFlow, DeepST, and conST) on the DLPFC dataset. The evaluation is organized into three categories: (i) Accuracy, assessed using Adjusted Rand Index (ARI), Normalized Mutual Information (NMI), Homogeneity (HOM), and Completeness (CM) scores; (ii) Continuity, evaluated with PAS and Average Silhouette Width (AWS) scores; and (iii) Efficiency, measured in terms of computation time and memory usage (SPACE). Each colored bar corresponds to one of the 12 DLPFC sections (151507–151676), enabling cross-section comparison of performance across methods.

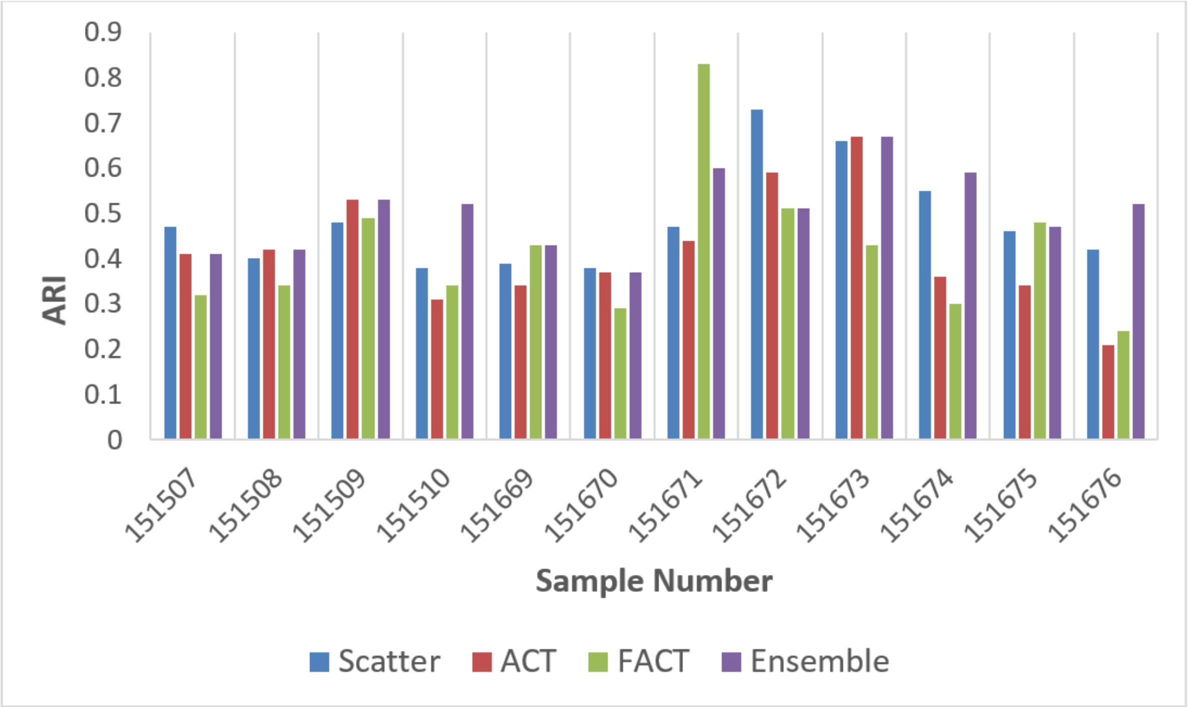


**Fig. 2.** Comparison of ARI scores across different variants of the proposed framework on DLPFC datasets

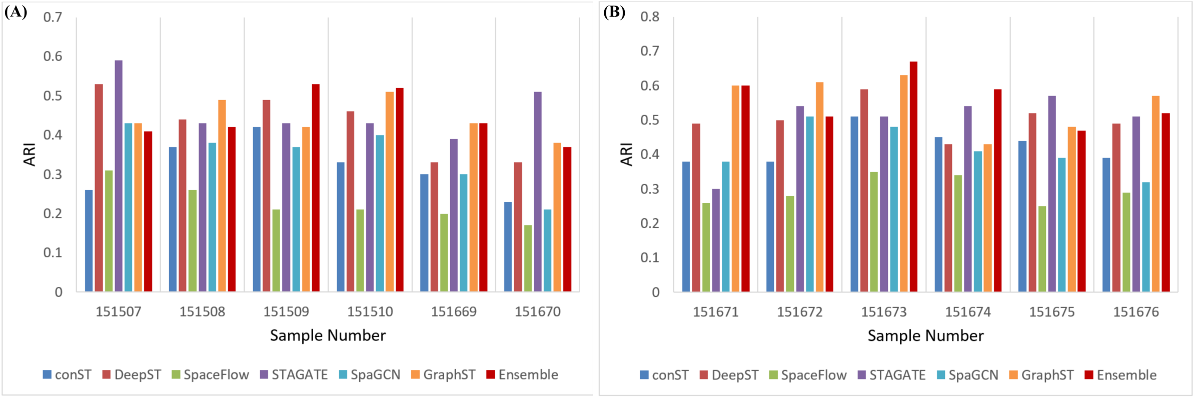


**Fig. 3.** Comparison of ARI scores across different state-of-the-arts method and proposed framework on DLPFC datasets

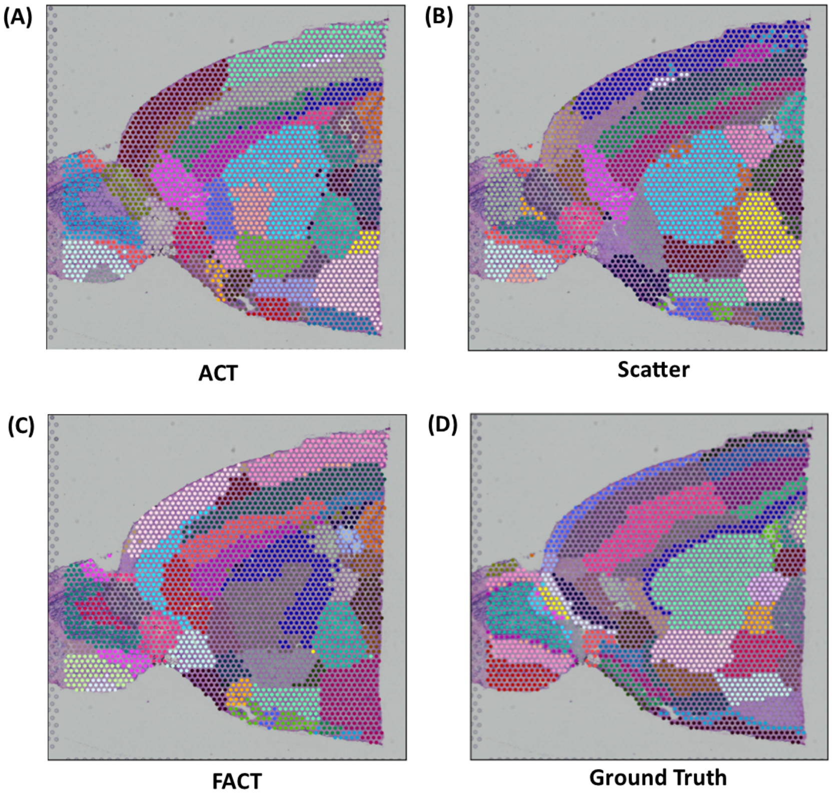


**Fig. 4.** Visual comparison of clustering performance across proposed approaches for 52 clusters of Mouse Brain Anterior dataset.

## Fine grained Mouse Brain Anterior Result Analysis

We have done finer analysis for Mouse Brain Anterior dataset, with trying to find out all 52 clusters. The Mouse Brain Anterior Visium section contains several distinct anatomical motifs that are useful for evaluating spatial clustering. In the ground truth, the left side corresponds to the rostral olfactory system, the dorsal surface contains ribbon-like cortical domains, the center contains a large compact deep forebrain territory, and the ventral region contains several adjacent but biologically distinct basal forebrain and olfactory-associated structures. Therefore, a biologically meaningful clustering method should recover both long cortical ribbons and compact nucleus-like domains rather than overemphasizing only one of these organizational patterns.

*ACT*

ACT provides the most biologically convincing overall reconstruction (Figure 4(A)). Quantitatively, it achieves the best performance among the three methods, with ARI = 0.4579, NMI = 0.7149, homogeneity = 0.7289, completeness = 0.7013, Moran's $I = 0.5236$, and Geary's $C = 0.4736$. Visually, ACT preserves the separation between the left olfactory compartment and the main cerebrum, reconstructs curved dorsal bands that are consistent with cortical lamination, and at the same time keeps the large central deep region compact while still subdividing the ventral forebrain into several coherent domains. This is biologically important because the anterior mouse brain is not purely layered tissue: the dorsal cortex is laminar, but the ventral and deep forebrain are organized as neighboring nuclei and compartments. ACT captures this dual organization better than the other methods.

The main limitation of ACT is mild over-smoothing in some dorsal outer regions, where thin cortical or orbital bands that appear distinct in the ground truth are merged into broader arcs. However, this is still a biologically reasonable error because the resulting domains remain spatially coherent and anatomically interpretable. Overall, ACT best preserves the hierarchical structure of the tissue by recovering both cortex-like ribbon organization and nucleus-like subcortical organization.

*FACT*

FACT achieves ARI = 0.4099, NMI = 0.7131, homogeneity = 0.7235, completeness = 0.7030, Moran's $I = 0.5232$, and Geary's $C = 0.4754$. Its strongest visual feature is the dorsal cortical pattern: the upper part of the section is organized into long, smooth, nested bands that look highly laminar (Figure 4(C)). From a cortical perspective, FACT is therefore attractive and appears to capture broad spatial gradients very clearly.

However, the deeper and ventral tissue is less biologically convincing. The central interior becomes too dominant as a broad merged region, and several ventral and right-lateral territories appear collapsed into larger compartments than expected from the ground truth. This suggests that FACT emphasizes the major cortical gradient but sacrifices some of the finer subcortical specificity. In biological terms, FACT seems to privilege laminar organization over regional heterogeneity in the deep forebrain. Thus, although FACT is strong at recovering broad dorsal organization, it is weaker at distinguishing nearby ventral and subcortical structures that should remain separate.

*Scatter*

Scatter achieves ARI = 0.4242, NMI = 0.7052, homogeneity = 0.7215, completeness = 0.6896, Moran's $I = 0.5215$, and Geary's $C = 0.4794$. Qualitatively, Scatter provides the cleanest coarse partition of the tissue. It separates the left olfactory region from the main forebrain, produces a large coherent central deep domain, and yields smooth and spatially compact regions with relatively little local fragmentation (Figure 4)(B). Biologically, this makes Scatter very plausible at the macro-anatomical level.

The main weakness of Scatter is under-resolution. The dorsal cortical mantle is represented by broad ribbons rather than finer area-plus-layer structure, and several ventral compartments are absorbed into larger domains. As a result, Scatter is biologically meaningful for recovering major tissue compartments, but it does not resolve the fine-grained anatomy as effectively as ACT. In other words, Scatter performs well for coarse atlas-like segmentation but is less effective for detailed recovery of the higher-resolution annotation.

## Fine grained Comparison with Ground Truth

The ground truth (Figure 4(D)) in this section contains both layered cortex and heterogeneous basal forebrain, so the best method is the one that balances these two anatomical principles. In this respect, ACT performs best. It preserves the dorsal ribbon-like cortical organization while also maintaining coherent and distinct subcortical and ventral domains. Scatter is the next best method because it recovers the large-scale anatomical structure cleanly and smoothly, but it tends to merge finer boundaries. FACT is the least convincing overall because, although it shows strong cortical ribboning, it appears to over-merge deep and ventral territories.

A useful biological interpretation is that the three methods emphasize different scales of organization. FACT is strongest for cortical laminar appearance, Scatter is strongest for smooth coarse regional partitioning, and ACT provides the best whole-section neuroanatomical realism. ACT is therefore the most suitable method if the goal is to recover anatomically meaningful fine spatial domains across the entire anterior mouse brain section.

*Overall Results for fine grained analysis for mouse brain anterior*

Based on both the quantitative metrics and the spatial plots, the overall ranking is

$$\text{ACT} > \text{Scatter} > \text{FACT}.$$

The main biological reason for this ranking is that ACT best respects the mixed anatomical organization of the tissue: it captures a layered dorsal cortical mantle together with compact and heterogeneous deep/ventral forebrain compartments. Scatter remains biologically interpretable and robust at a coarse scale, whereas FACT appears to overemphasize broad laminar structure at the expense of subcortical specificity.

ACT best preserves the hierarchical anatomical organization of the anterior mouse brain, recovering a layered dorsal cortical mantle together with compact deep and ventral forebrain compartments. FACT accentuates laminar cortical

**Table 10.** Multi-dimensional performance comparison for 52-cluster Mouse Brain Anterior dataset. Stars indicate relative performance across biological and quantitative criteria.

| Criterion | ACT | Scatter | FACT |
|---|---|---|---|
| Clustering Accuracy (ARI/NMI) | ⋆⋆⋆⋆⋆ | ⋆⋆⋆⋆ | ⋆⋆⋆⋆ |
| Spatial Autocorrelation | ⋆⋆⋆⋆⋆ | ⋆⋆⋆⋆ | ⋆⋆⋆⋆ |
| Cortical Ribbon Representation | ⋆⋆⋆⋆ | ⋆⋆⋆ | ⋆⋆⋆⋆⋆ |
| Subcortical Resolution | ⋆⋆⋆⋆⋆ | ⋆⋆⋆ | ⋆⋆ |
| Fine-grained Domain Recovery | ⋆⋆⋆⋆⋆ | ⋆⋆⋆ | ⋆⋆ |
| Macro-anatomical Coherence | ⋆⋆⋆⋆⋆ | ⋆⋆⋆⋆⋆ | ⋆⋆⋆ |
| **Overall Biological Fidelity** | ⋆⋆⋆⋆⋆ | ⋆⋆⋆⋆ | ⋆⋆⋆ |

**Table 11.** Quantitative performance metrics for clustering.

| Method | ARI | NMI | Homogeneity | Completeness | Moran's I | Geary's C |
|---|---|---|---|---|---|---|
| ACT | **0.4579** | **0.7149** | **0.7289** | 0.7013 | **0.5236** | **0.4736** |
| Scatter | 0.4242 | 0.7052 | 0.7215 | 0.6896 | 0.5215 | 0.4794 |
| FACT | 0.4099 | 0.7131 | 0.7235 | **0.7030** | 0.5232 | 0.4754 |

structure but over-merges subcortical territories, whereas Scatter yields smooth and coherent macro-domains at the cost of fine anatomical resolution. Consequently, ACT provides the most biologically meaningful overall clustering for this section.

## References

Fatima Batool and Christian Hennig. Clustering with the average silhouette width. *Computational Statistics & Data Analysis*, 158:107190, 2021. doi:10.1016/j.csda.2021.107190.

R. C. Geary. The contiguity ratio and statistical mapping. *The Incorporated Statistician*, 5(3):115–146, 1954. doi:10.2307/2986645.

Yunfei Hu, Manfei Xie, Yikang Li, Mingxing Rao, Wenjun Shen, Can Luo, Haoran Qin, Jihoon Baek, and Maizie Xin Zhou. Benchmarking clustering, alignment, and integration methods for spatial transcriptomics. *Genome Biology*, 25:212, 2024. doi:10.1186/s13059-024-03361-0.

P. A. P. Moran. Notes on continuous stochastic phenomena. *Biometrika*, 37(1–2):17–23, 1950. doi:10.1093/biomet/37.1-2.17.

Anupriya Vysala and Joseph Gomes. Evaluating and validating cluster results. *arXiv preprint arXiv:2007.08034*, 2020.
